# Supplementary material for: Bistable Bacterial Growth Dynamics in the Presence of Antimicrobial Agents
Source: Antibiotics (Basel). 2021 Jan 18;10(1):87. doi: 10.3390/antibiotics10010087 (PMC7831100; doi:10.3390/antibiotics10010087)
Supplement: Supplementary file 1 [file antibiotics-10-00087-s001.pdf]

Bistable bacterial growth dynamics in the presence of antimicrobial agents – supplementary material

We first provide the technical details needed to produce the growth curve and final capacity figures we used in the article (Figure S1). Then we present some experimental results regarding antibiotic and peptide stability (Figures S2-S3). Finally, we enclose all the experimental results for each performed experiment (Figures S4-S7).

Data Processing:

Growth curves: If the final OD reading is below a threshold (for the LB medium, ODthresh=0.35, for the Minimal medium ODthresh=0.18, for the Robot data ODthresh=0.5), the well is defined to have no growth. If there is growth, the growth curves (Figures 2B,D, 3B,4B, 5B, 7C) are found by the following algorithm: a) Smooth the data (by the MATLAb program smooth which uses a moving average filter over nsmooth=3 terms), b) Cut the data points which are below the OD reader resolved region (ODcut=0.11) c) To clear the oscillation pass the maximal capacity, set all data points after the maximal value of the smoothed data to this maximal value. d) Ignore the data points which have a slope which is small (smaller than half the averaged slope of the data). e) Compute the growth function for the smoothed, cut data : ODsm(t+dt)-ODsm(t)/dt and plot it versus the corresponding mid-point value ((ODsm(t)+ODsm(t+dt))/2).

Figure S1 examines the dependency of the above algorithm on the number nsmooth of terms in the smoothing filter and demonstrates that the maximal capacity hardly depends on nsmooth whereas the maximal growth rate depends on nsmooth.

Maximal capacity : If there is growth, and if the growth is complete in the experiment time frame, the value of the maximal capacity (Figures 3C,D, 4C,D, 5C-F,7D,E) is defined to be the maximal smooth OD value (last point on the solid growth curve), normalized by the mean of the control maximal smooth OD values. The growth is called completed, either when the cutting time is prior to the last possible one (one before last reading) or if the OD value is sufficiently large (larger than the control maximal capacity mean minus 3 standard deviations of the control maximal capacity data). We notice that the Maximal capacity produces a more robust criteria than the

maximal growth rate, which is defined as the maximal values of the OD growth function divided by the OD value: $\text{Rmax} = \text{Max\_t}\ \{2*[\text{ODsm}(t+dt)-\text{ODsm}(t)]/[dt((\text{ODsm}(t)+\text{ODsm}(t+dt))]\}$, see Figure S1D.

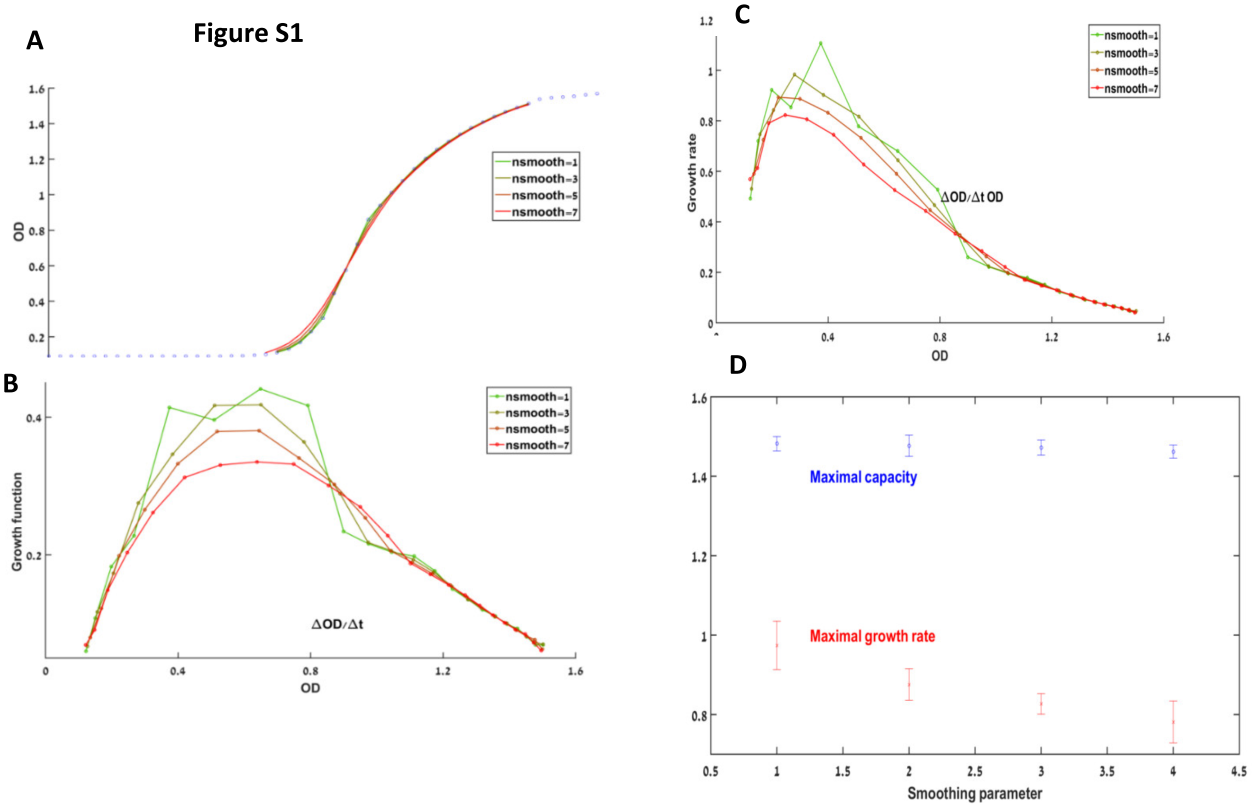

Figure S1: Robustness to smoothing: (A) Growth curve of one of the control wells: data points are in blue, and the solid curves are the curves achieved for increasing smoothing index (nsmooth=1,3,5,7). **(B)** The corresponding growth function dependence on nsmooth **(C)** The Growth rate for the same setup. **(D)** Mean and standard deviations of the maximal capacity and the maximal growth rate of all the control wells for increasing smoothing parameters. While the maximal capacity is not dramatically affected by smoothing, the maximal growth rate is, with an order of magnitude larger standard deviation.

Peptide loss of potency leads to BIK dynamics.

Figure S2 demonstrates that the dynamics displayed by bacteria in the presence of antimicrobial peptides is due to the peptides losing their ability to damage bacterial

membranes a very short time after the initial incubation. If few bacteria are present at that time, they are completely growth inhibited, but if there are enough bacteria, some can survive the initial encounter with the peptide and show undisturbed growth later on. Indeed, comparing fresh Polymixin B (PMB), PMB that was incubated for an hour in LB media prior to usage, or PMB that was incubated for an hour with 10^6 cfu bacteria in LB (and then centrifuged to discard the bacteria), the 3 types of PMB give different inhibitory effects over an inoculum of 10^5 bacteria. This is shown in a PMB concentration low enough for the amount that was "wasted" upon the previous encounter with bacteria to be significant. As can be seen in Figure S2, the least potent PMB is the one incubated for an hour with bacteria, and the most potent (that also inhibits bacterial growth completely in this PMB concentration) is the freshly added PMB.

Figure S2

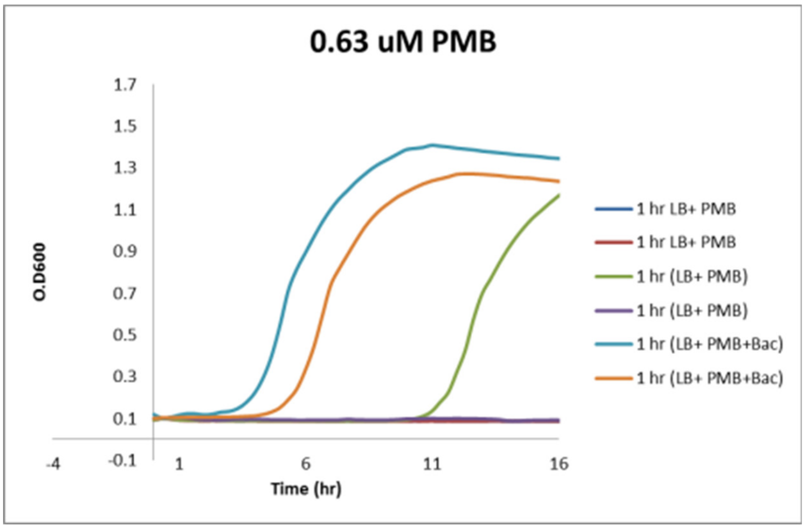

Figure S2. PMB potency after incubation with bacteria. Bacteria were grown in different media as indicated for 16h and their OD_{600} was monitored in a microplate reader every 20 min. Bacteria grown in LB with PMB that was previously incubated with bacteria grow to full capacity, bacteria grown in LB with PMB that was previously incubated in 37°C grow late, and bacteria grown in LB with fresh PMB fail to grow (for each condition duplicates are shown).

Figure S3 shows that the demonstrates that no concentration of Ampicillin showed the same kind of extinction – in all settings its effect remained fairly potent.

Figure S3

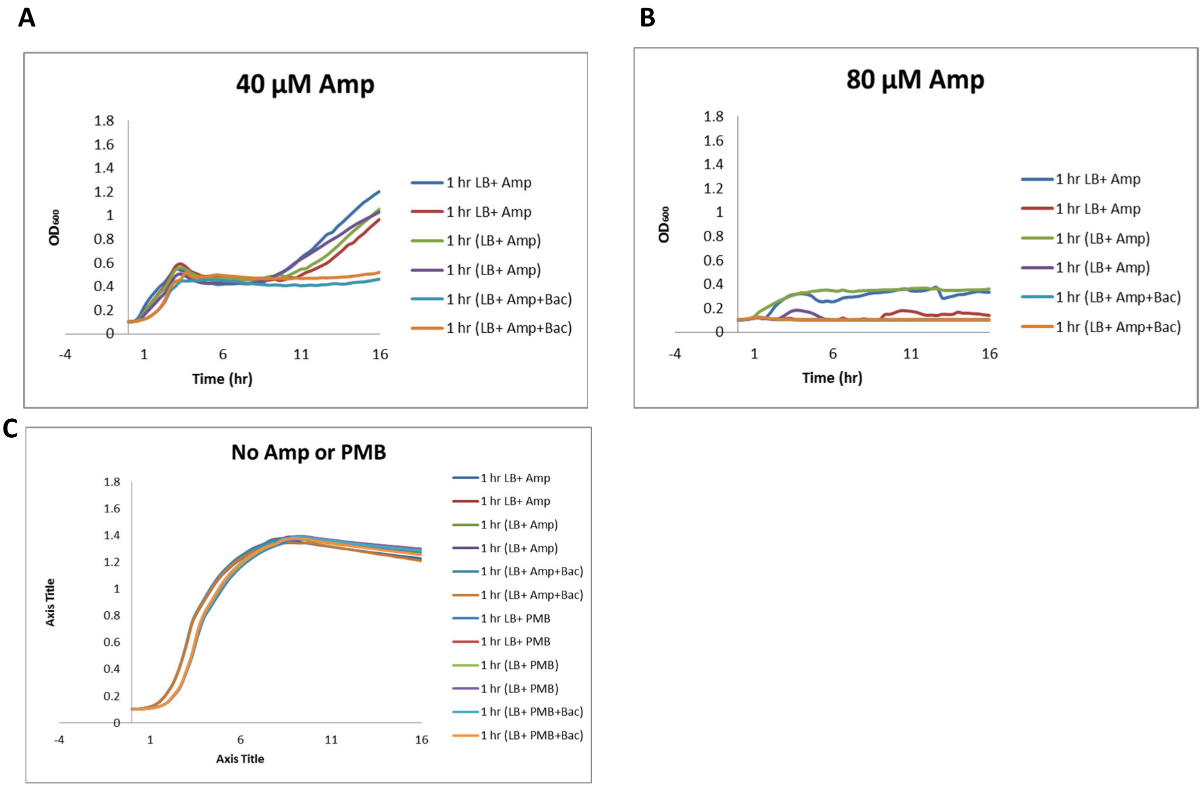

Figure S3. Amp potency after incubation with bacteria. (A), (B) Bacteria grown in different concentrations of Amp that was either previously incubated in heat or with bacteria, grow with similar dynamics to bacteria that were grown with fresh Amp. **(C)** Control plates for the experiments shown in S2 and S3A,B show identical growth curves.

Bacteria grown in the presence of commercial bacteriostatic antibiotics exhibit bi-stability and A-dependent dynamics (BAD)

<u>Comment:</u> Experiments were performed in two distinct plate readers. Often, results from one of the readers contained a distinct growth output till an OD_{600} of about 0.3. This growth is probably a result of technical issues in the said plate reader and didn't change the trends of the results.

Below we present growth curves, growth functions and maximal capacity of all experiments performed with bacteriostatic antibiotics (Chloramphenicol and Tetracycline – Figures S4A and S4C), and the maximal capacity of 7 experiments with Chloramphenicol together (Figure S4B).

Figures S4A : Chloramphenicol individual experiments

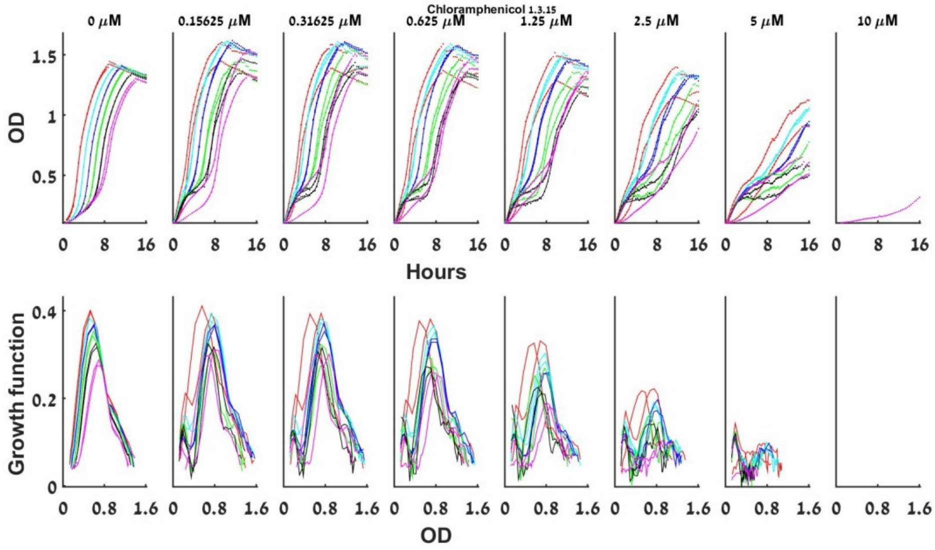

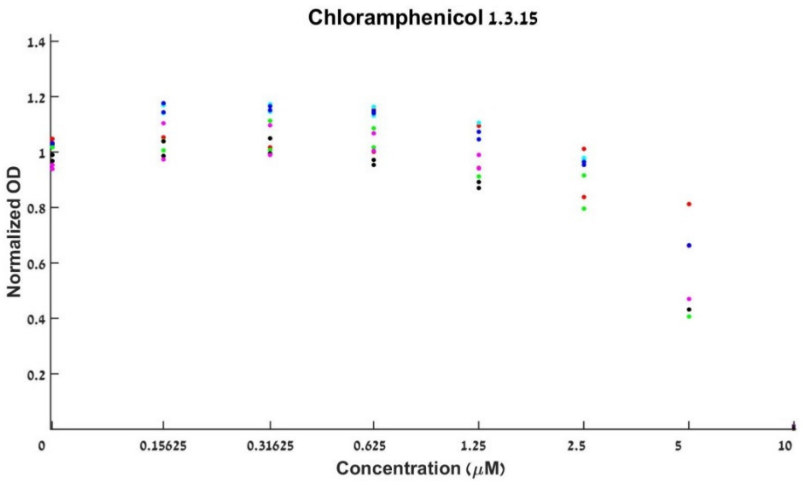

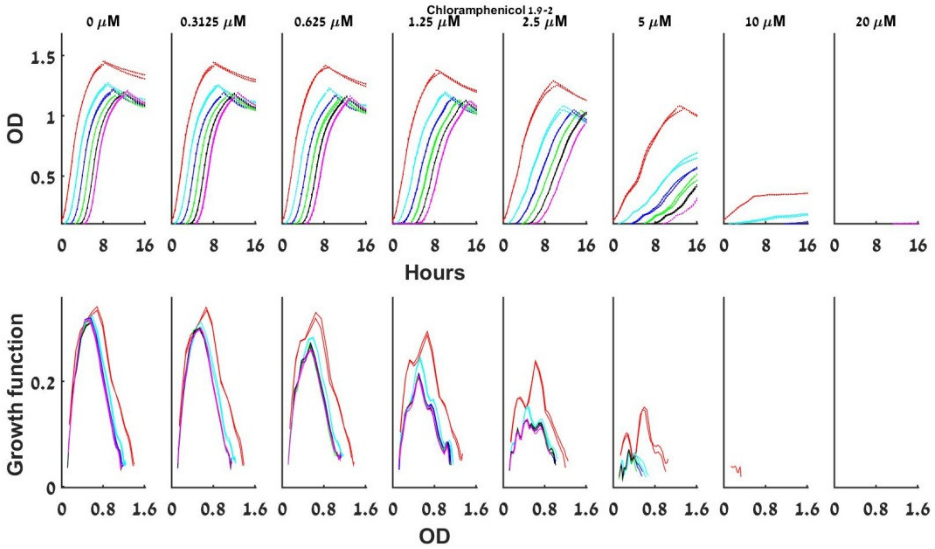
Chloramphenicol 1.9-2
0 μM
0.3125 μM
0.625 μM
1.25 μM
2.5 μM
5 μM
10 μM
20 μM
OD
Hours
Growth function
OD

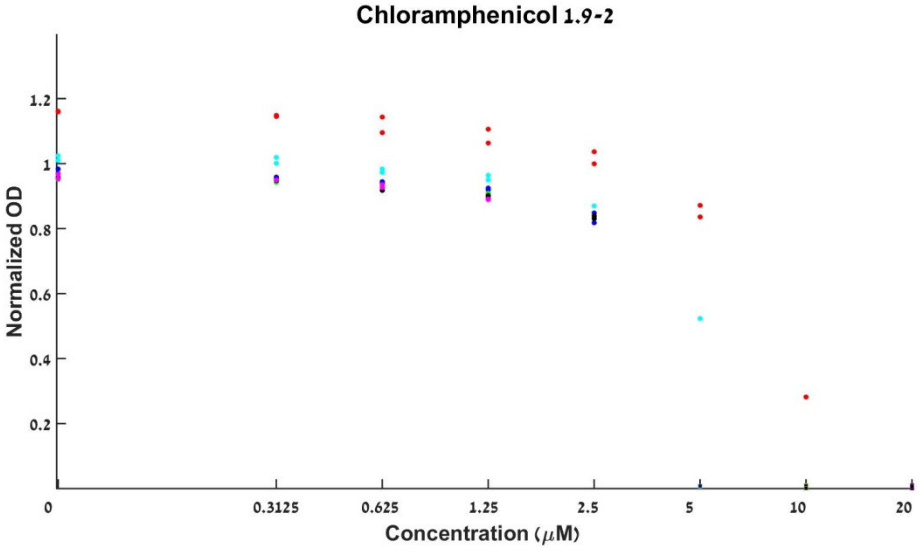
Chloramphenicol 1.9-2
Normalized OD
Concentration (μM)

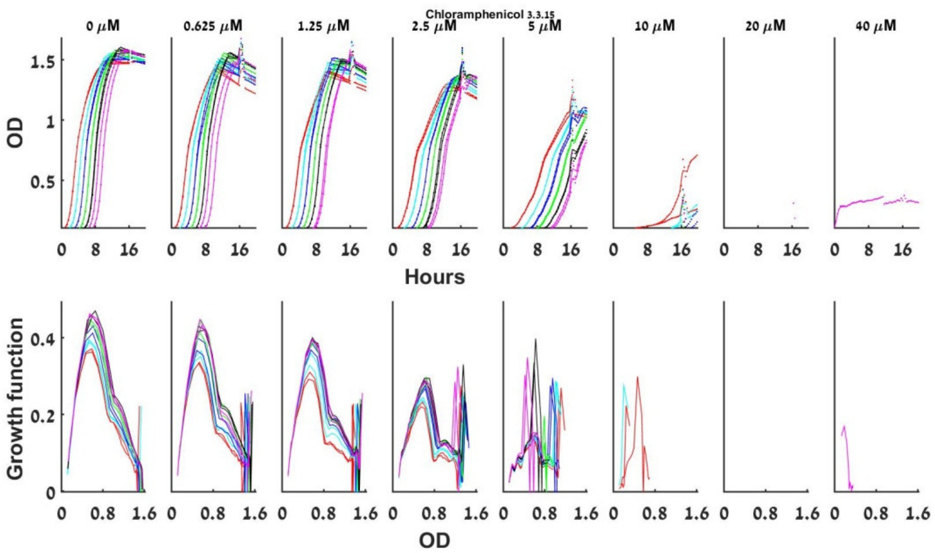
Chloramphenicol 3.3.15
0 μM
0.625 μM
1.25 μM
2.5 μM
5 μM
10 μM
20 μM
40 μM
OD
Hours
Growth function
OD

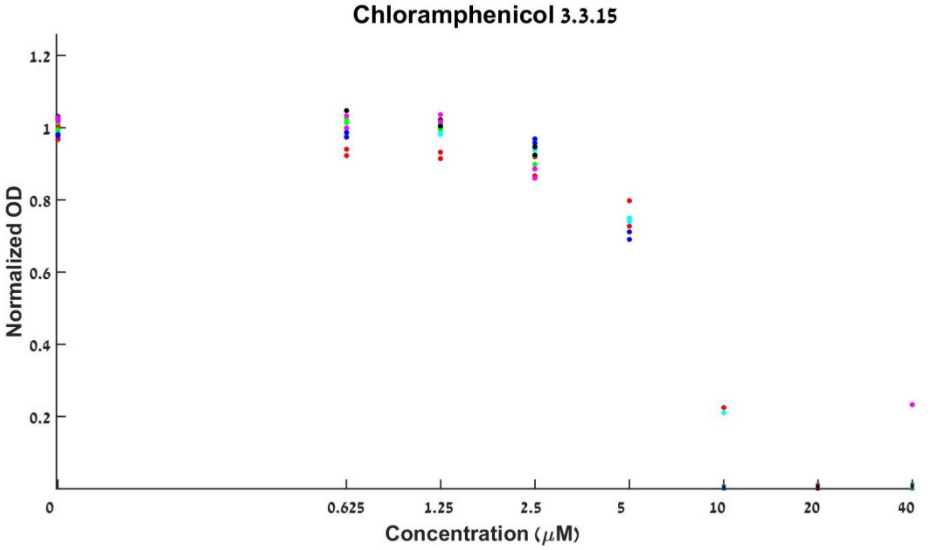
Chloramphenicol 3.3.15
Normalized OD
Concentration (μM)

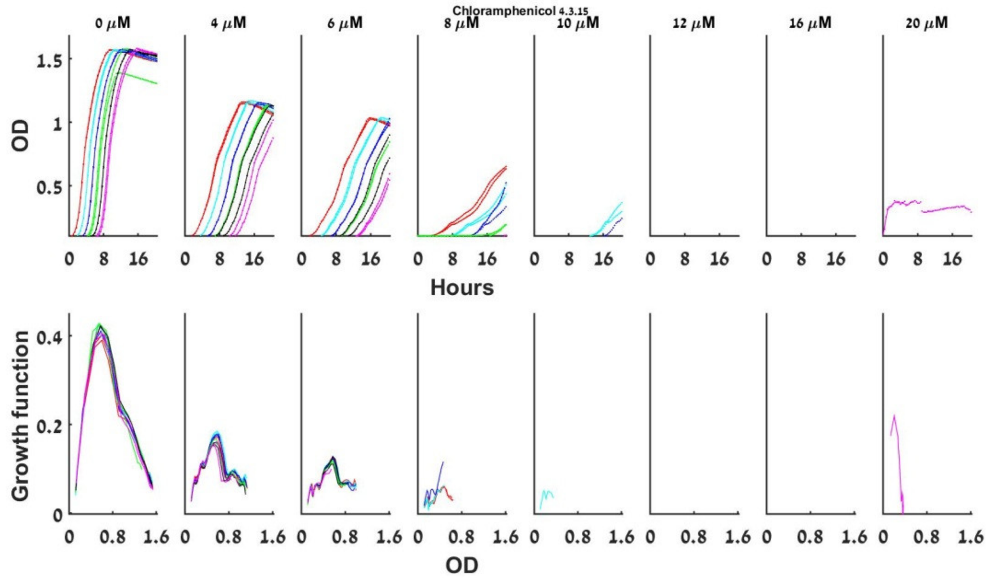
Chloramphenicol 4.3.15
0 μM
4 μM
6 μM
8 μM
10 μM
12 μM
16 μM
20 μM
OD
Hours
Growth function
OD

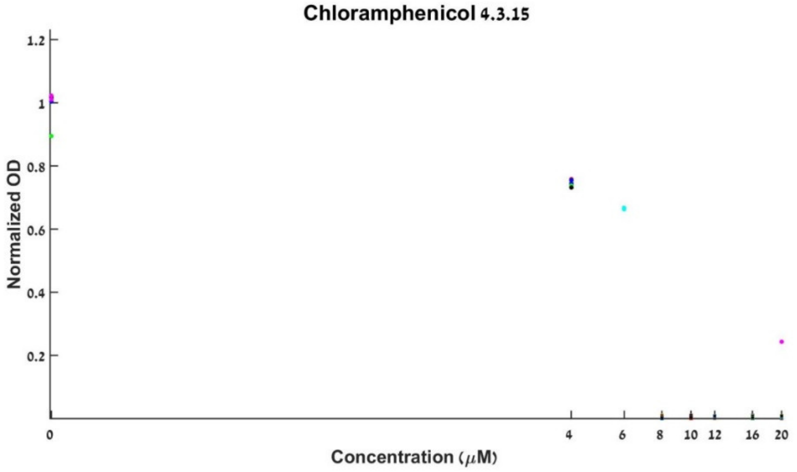
Chloramphenicol 4.3.15
Normalized OD
Concentration (μM)

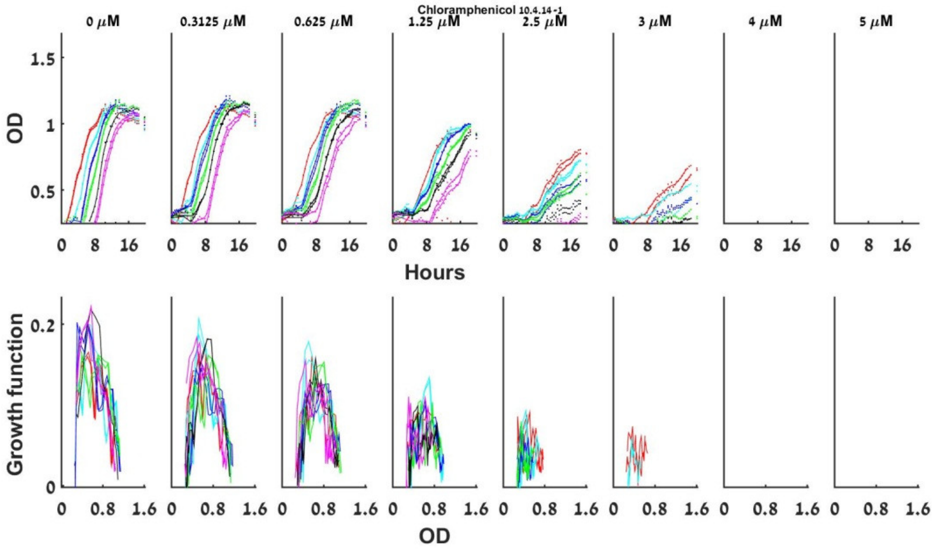
Chloramphenicol 10.4.14-1
0 μM
0.3125 μM
0.625 μM
1.25 μM
2.5 μM
3 μM
4 μM
5 μM
OD
Hours
Growth function
OD

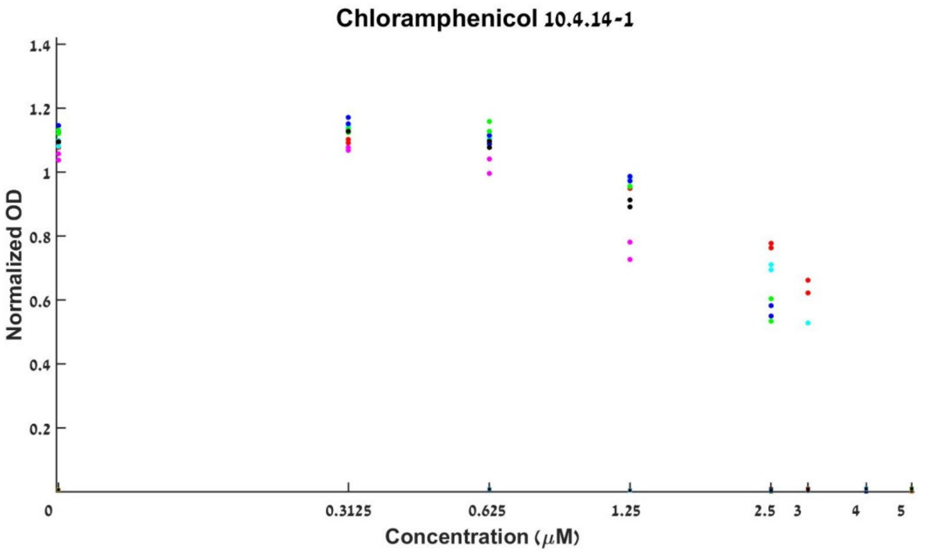

Chloramphenicol 10.4.14-1
Normalized OD
Concentration (μM)
0
0.3125
0.625
1.25
2.5
3
4
5

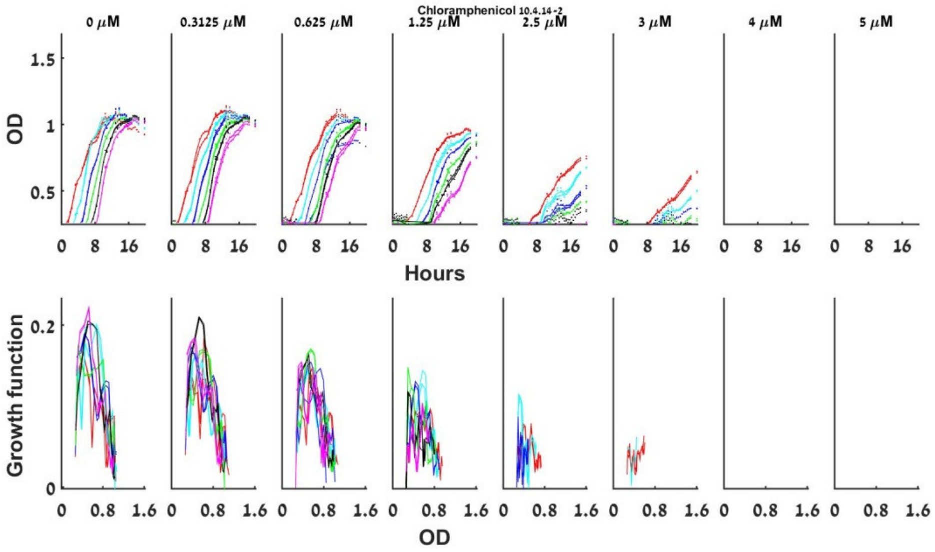

Chloramphenicol 10.4.14-2
0 μM
0.3125 μM
0.625 μM
1.25 μM
2.5 μM
3 μM
4 μM
5 μM
OD
Hours
Growth function
OD

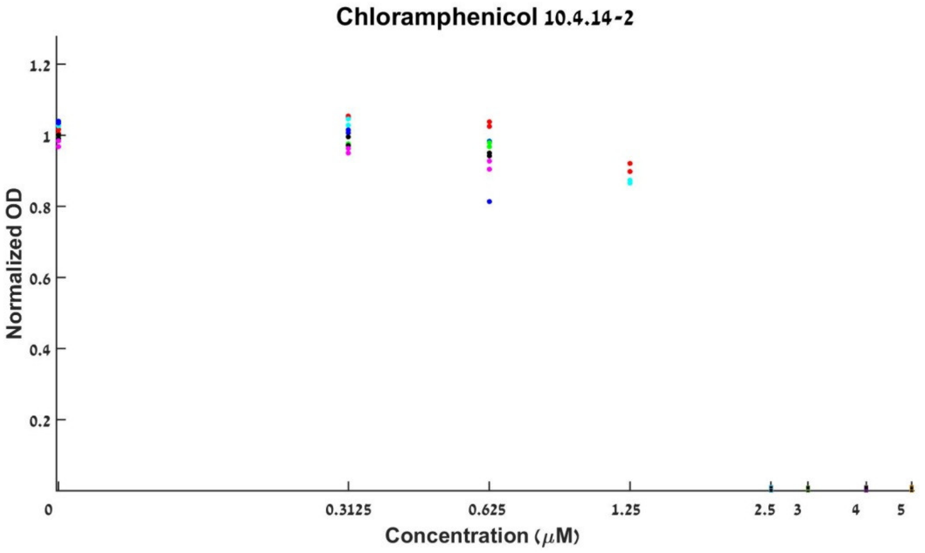

Chloramphenicol 10.4.14-2
Normalized OD
Concentration (μM)
0
0.3125
0.625
1.25
2.5
3
4
5

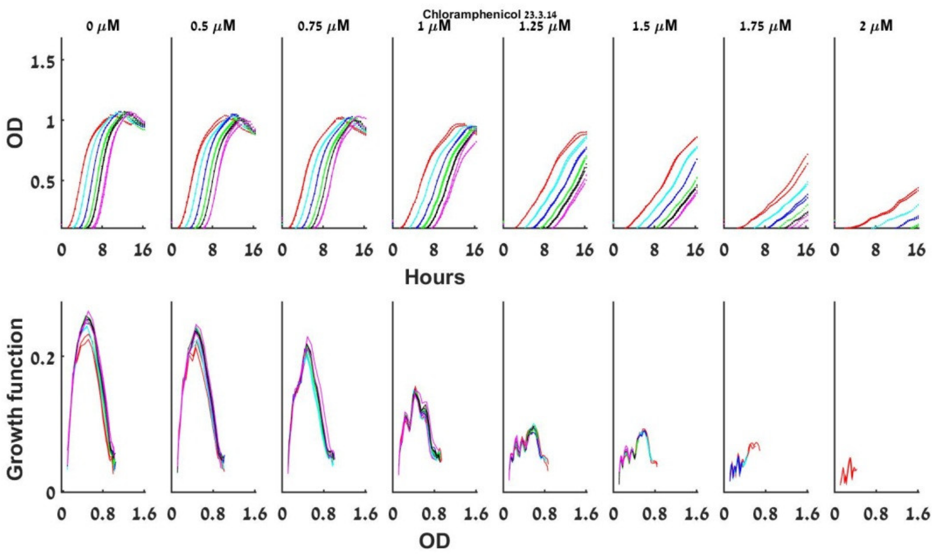
Chloramphenicol 23.3.14
0 μM
0.5 μM
0.75 μM
1 μM
1.25 μM
1.5 μM
1.75 μM
2 μM
OD
Hours
Growth function
OD

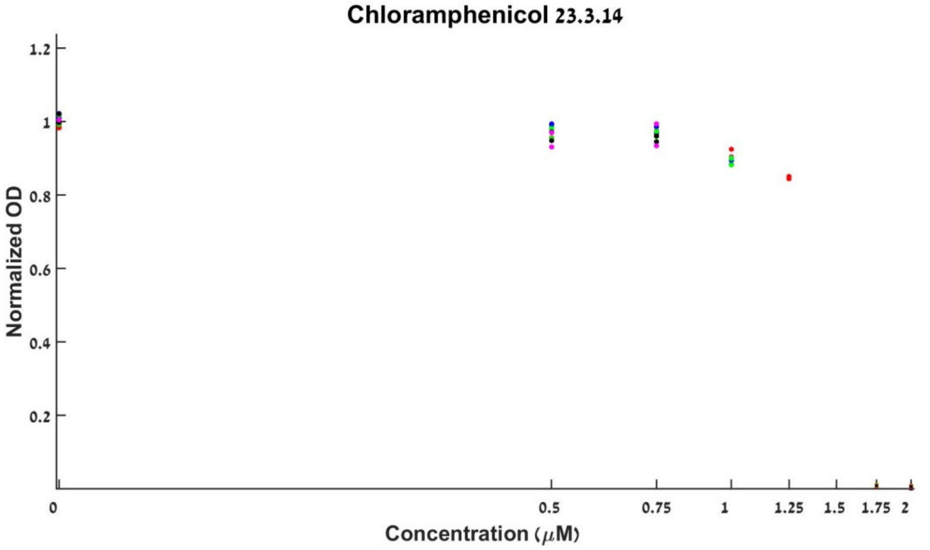
Chloramphenicol 23.3.14
Normalized OD
Concentration (μM)

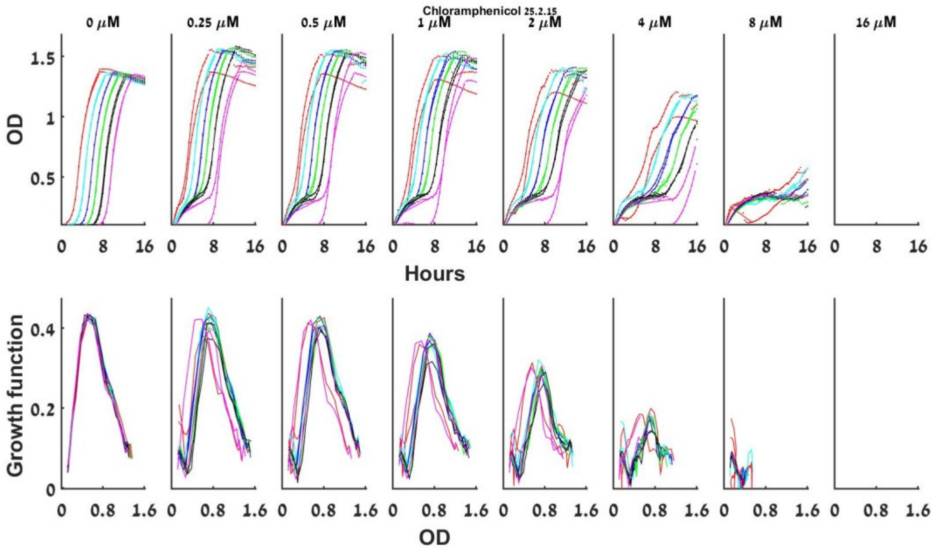
Chloramphenicol 25.2.15
0 μM
0.25 μM
0.5 μM
1 μM
2 μM
4 μM
8 μM
16 μM
OD
Hours
Growth function
OD

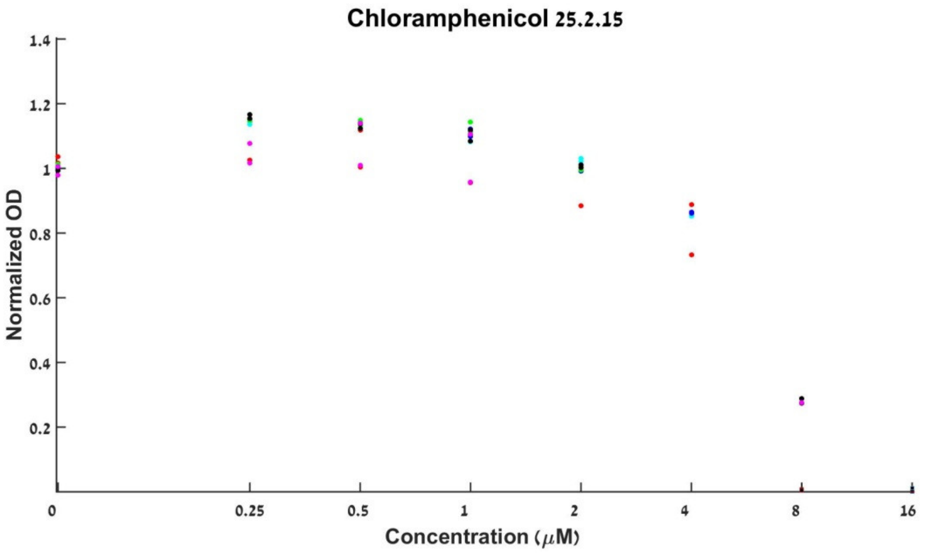

Figures S4B : Chloramphenicol 7 experiments combined

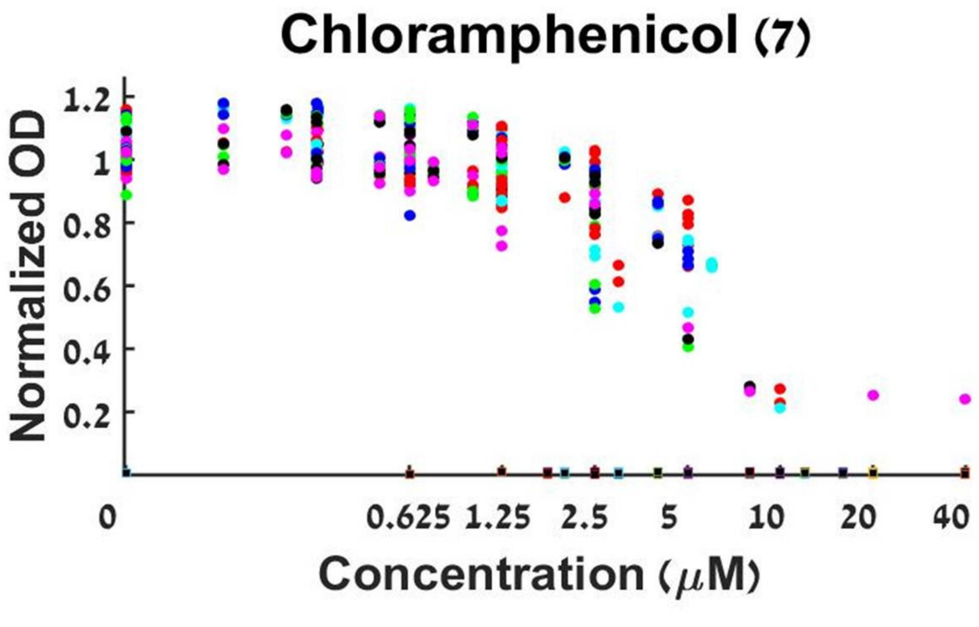

Figures S4C : Tetracycline individual experiments

C

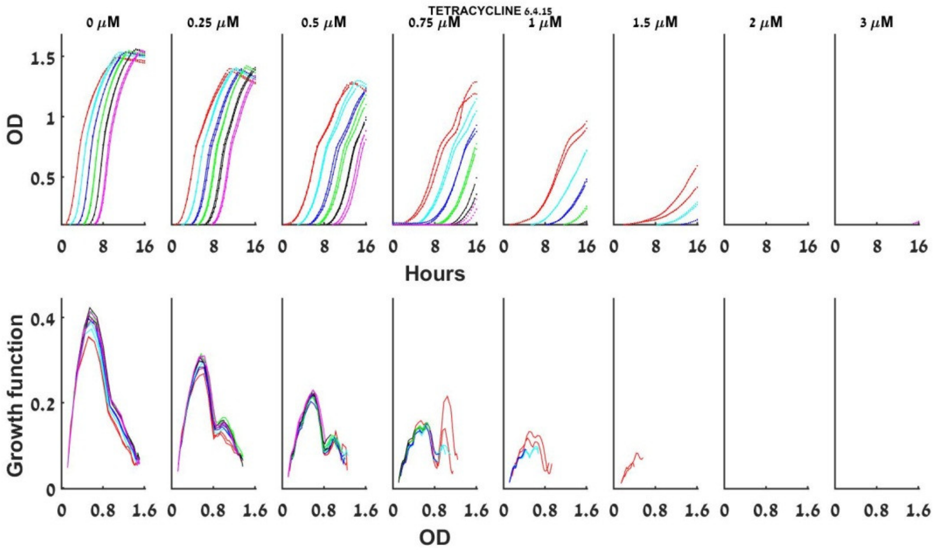

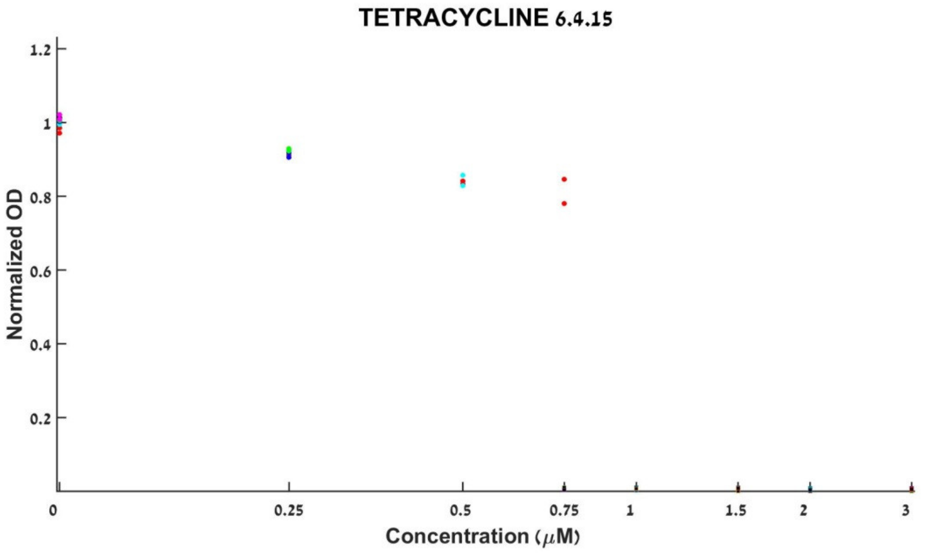

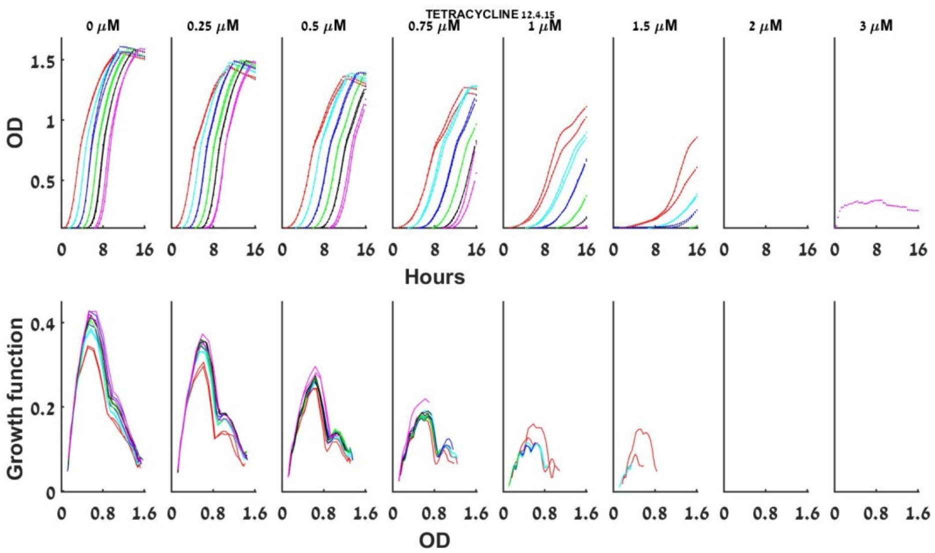
TETRACYCLINE 12.4.15
0 μM
0.25 μM
0.5 μM
0.75 μM
1 μM
1.5 μM
2 μM
3 μM
OD
Hours
Growth function
OD

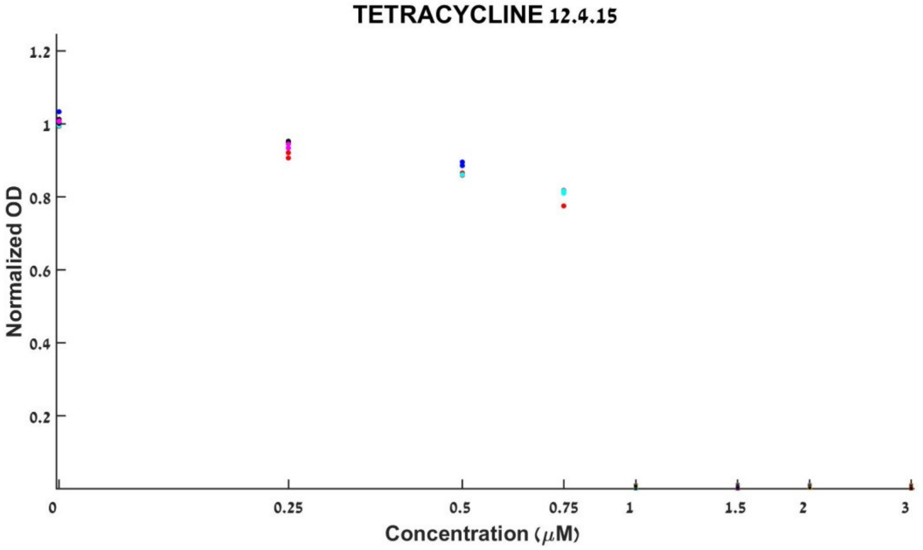
TETRACYCLINE 12.4.15
Normalized OD
Concentration (μM)

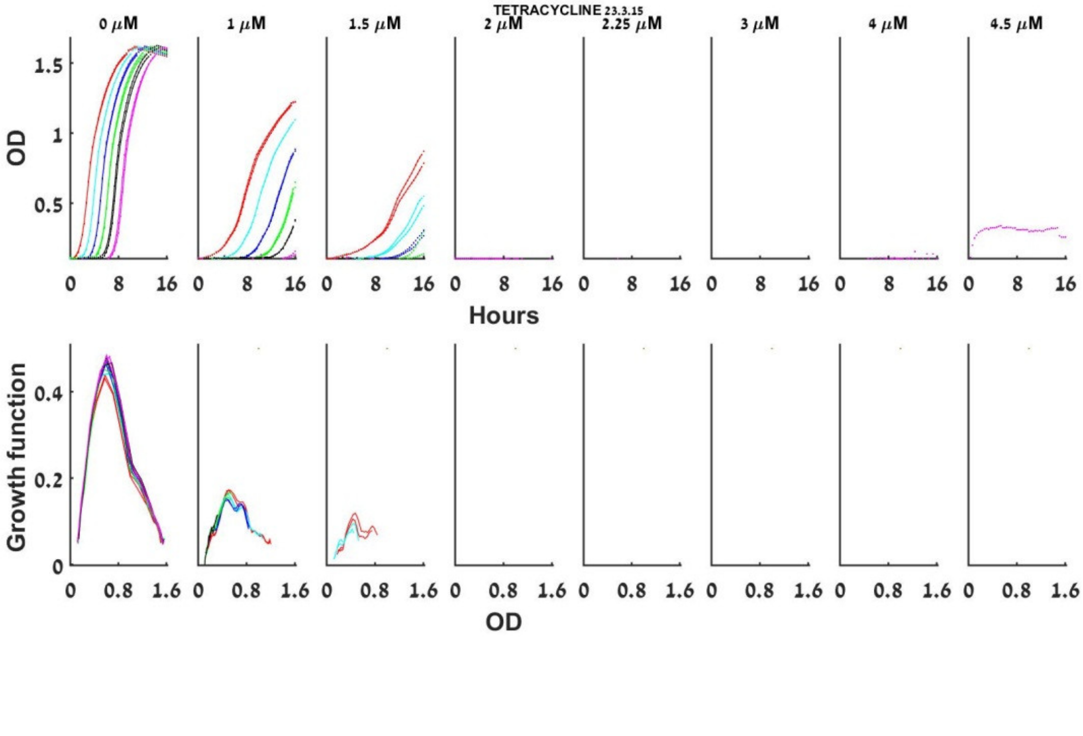
TETRACYCLINE 23.3.15
0 μM
1 μM
1.5 μM
2 μM
2.25 μM
3 μM
4 μM
4.5 μM
OD
Hours
Growth function
OD

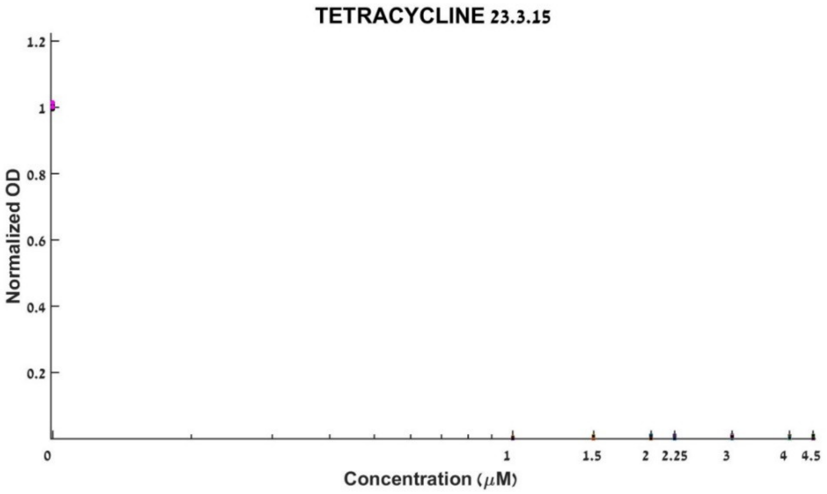
TETRACYCLINE 23.3.15
Normalized OD
1.2
1
0.8
0.6
0.4
0.2
0
1
1.5
2
2.25
3
4
4.5
Concentration (μM)

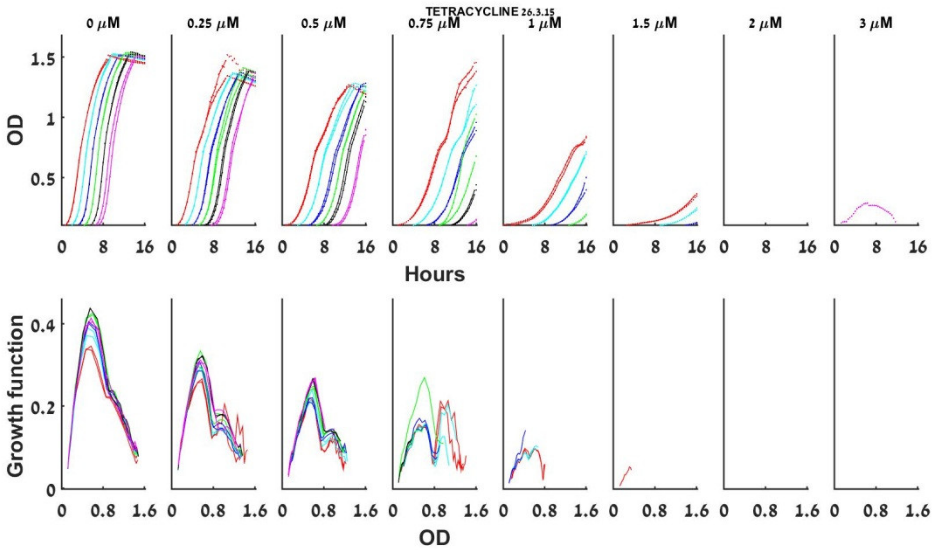
TETRACYCLINE 26.3.15
0 μM
0.25 μM
0.5 μM
0.75 μM
1 μM
1.5 μM
2 μM
3 μM
OD
1.5
1
0.5
0 8 16
Hours
Growth function
0.4
0.2
0
0 0.8 1.6
OD

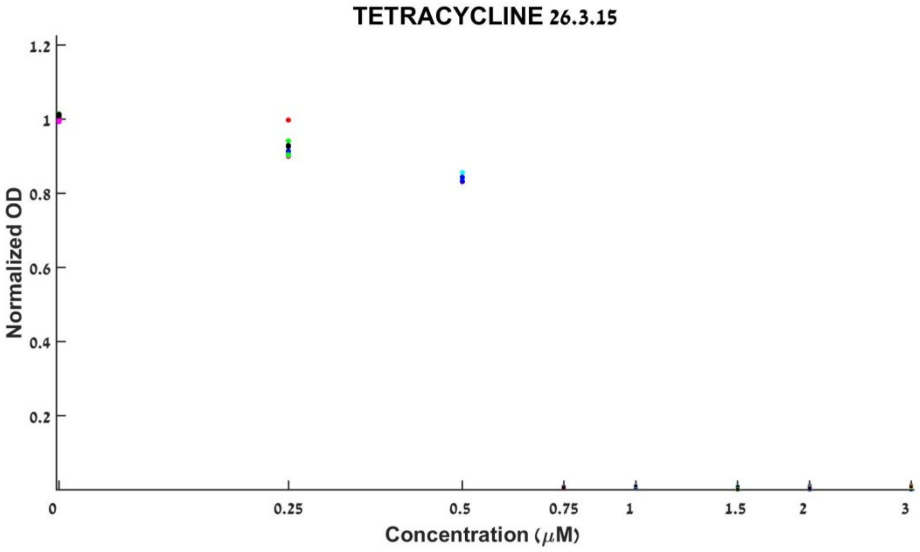
TETRACYCLINE 26.3.15
Normalized OD
1.2
1
0.8
0.6
0.4
0.2
0
0.25
0.5
0.75
1
1.5
2
3
Concentration (μM)

Figure S4. Dynamics of bacterial growth with Chloramphenicol and Tetracycline. Growth curves, growth functions and maximal capacity of single experiments performed with bacteriostatic antibiotics (**A**) Single IE experiments with Chloramphenicol. (**B**) Maximal capacity of 7 IE experiments with Chloramphenicol. (**C**) Single IE experiments with Tetracycline.

Bacteria grown in the presence of commercial bacteriolytic and bactericidal antibiotics exhibit bi-stability and BMFD dynamics

Growth curves, growth functions and maximal capacity of all experiments performed with bactericidal and bacteriolytic antibiotics (Gentamycin, Kanamycin, Ampicillin and Carbenicillin see Figure S5 A-D).

Figure S5A: Gentamycin individual experiments

A

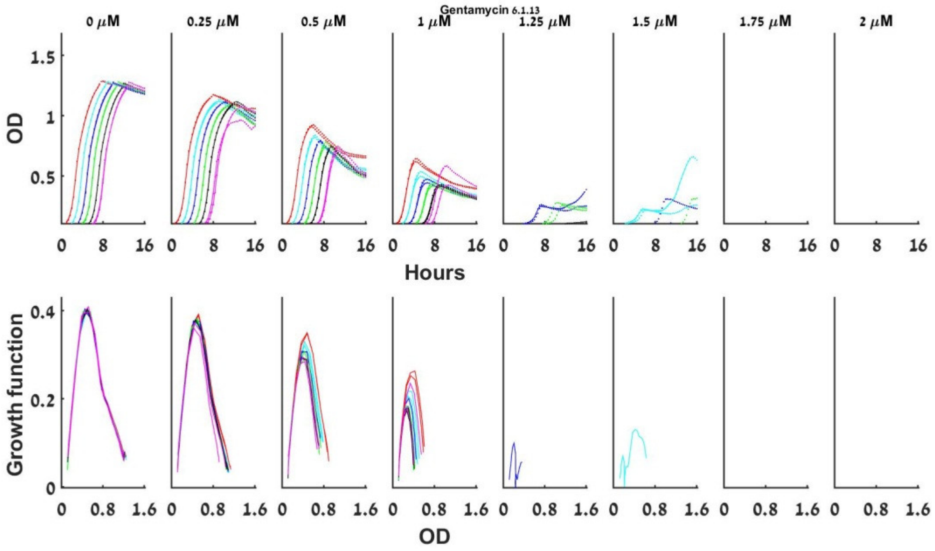

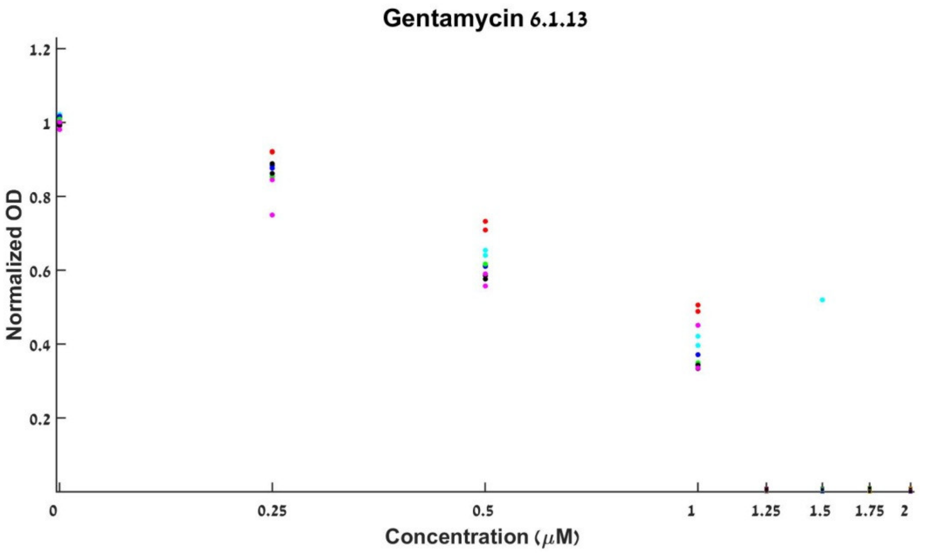
Gentamycin 6.1.13
Normalized OD
1.2
1
0.8
0.6
0.4
0.2
0
0.25
0.5
1
1.25
1.5
1.75
2
Concentration (μM)

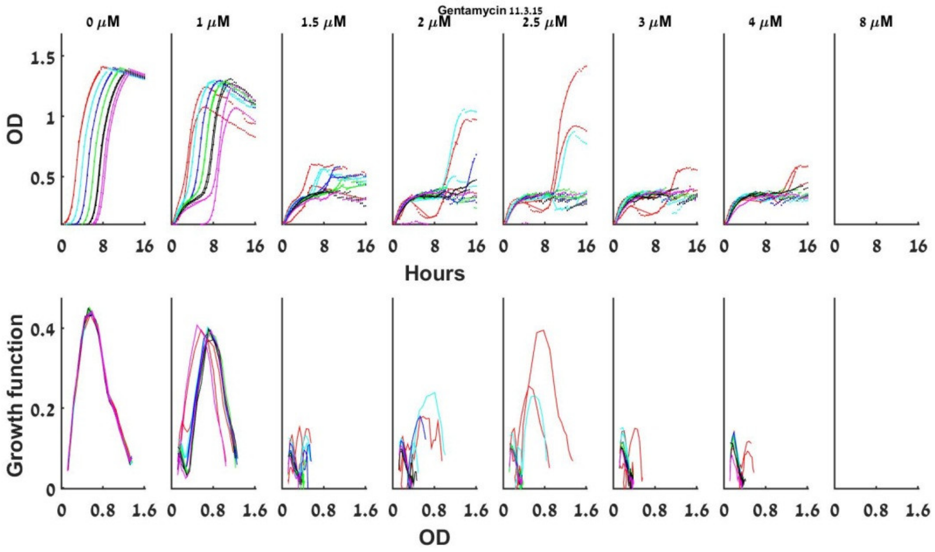
Gentamycin 11.3.15
0 μM
1 μM
1.5 μM
2 μM
2.5 μM
3 μM
4 μM
8 μM
OD
1.5
1
0.5
0
8
16
Hours
Growth function
0.4
0.2
0
0.8
1.6
OD

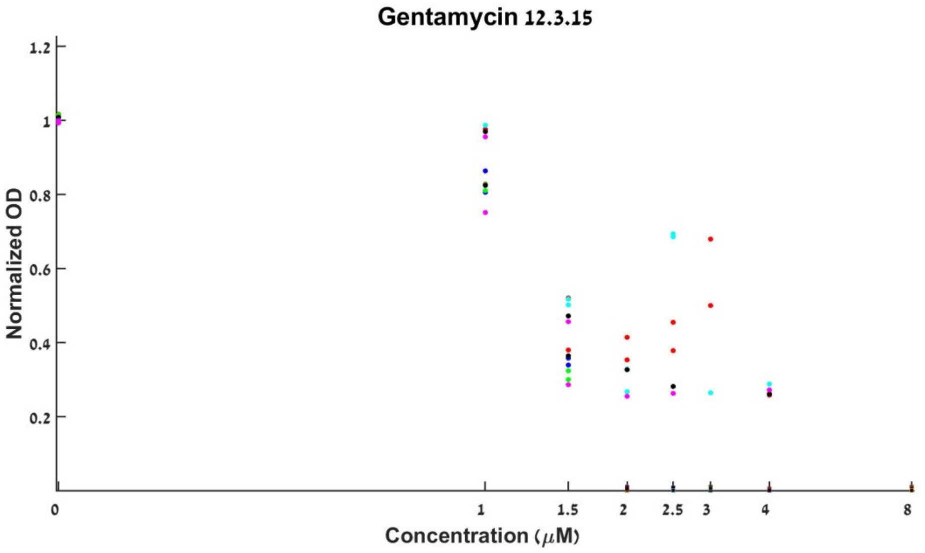
Gentamycin 12.3.15
Normalized OD
1.2
1
0.8
0.6
0.4
0.2
0
1
1.5
2
2.5
3
4
8
Concentration (μM)

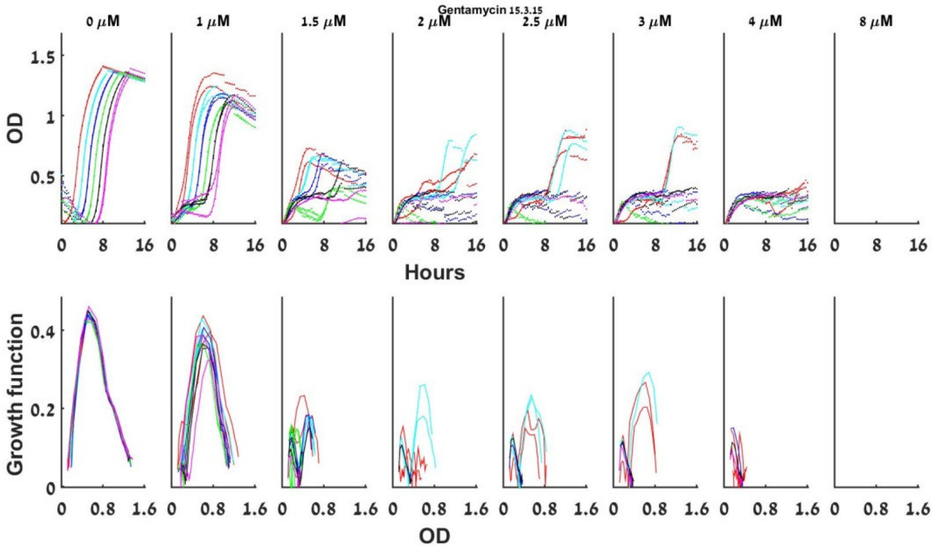
Gentamycin 15.3.15
0 μM
1 μM
1.5 μM
2 μM
2.5 μM
3 μM
4 μM
8 μM
OD
Hours
Growth function
OD

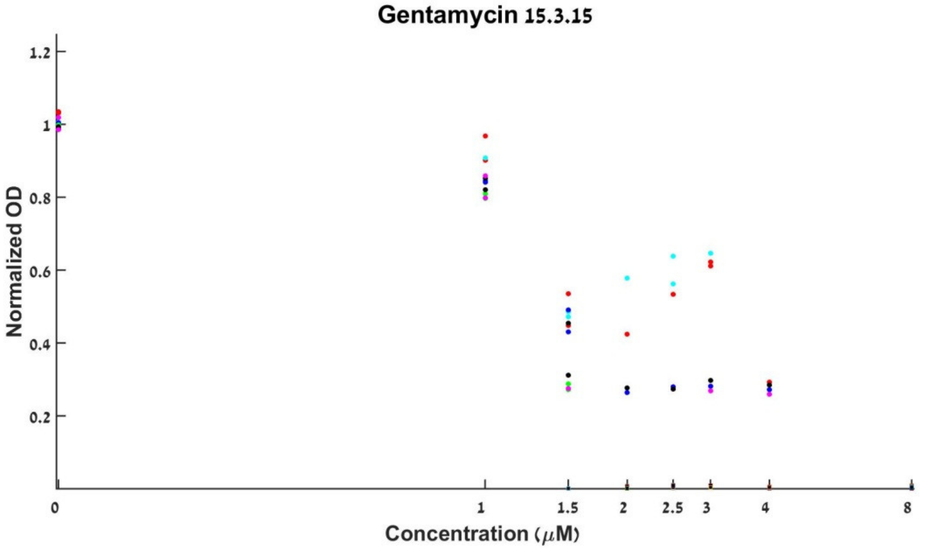
Gentamycin 15.3.15
Normalized OD
Concentration (μM)

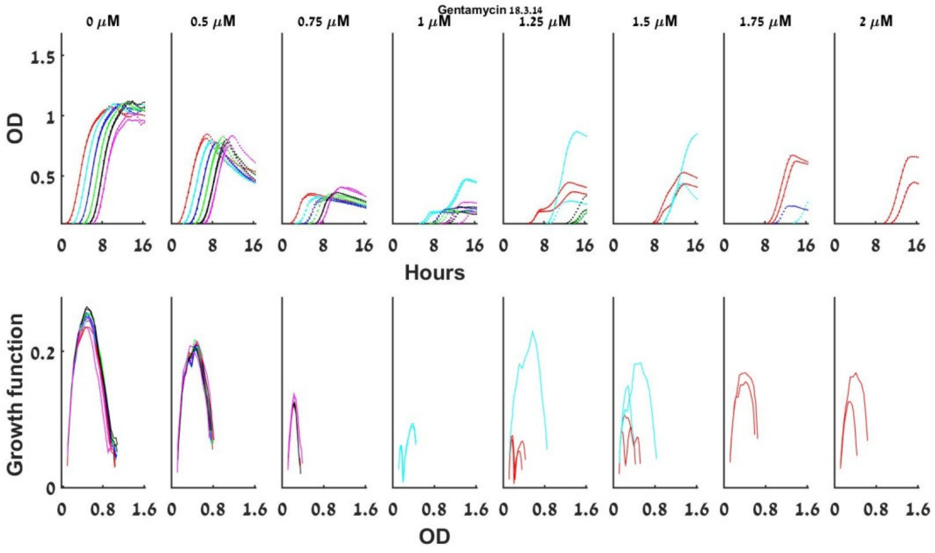
Gentamycin 18.3.14
0 μM
0.5 μM
0.75 μM
1 μM
1.25 μM
1.5 μM
1.75 μM
2 μM
OD
Hours
Growth function
OD

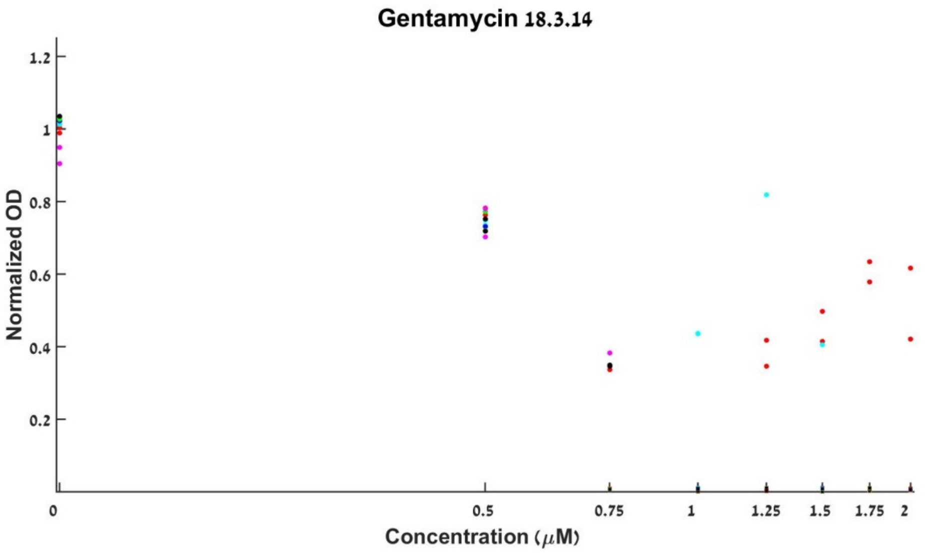

Gentamycin 18.3.14
Normalized OD
1.2
1
0.8
0.6
0.4
0.2
0
0.5
0.75
1
1.25
1.5
1.75
2
Concentration (μM)

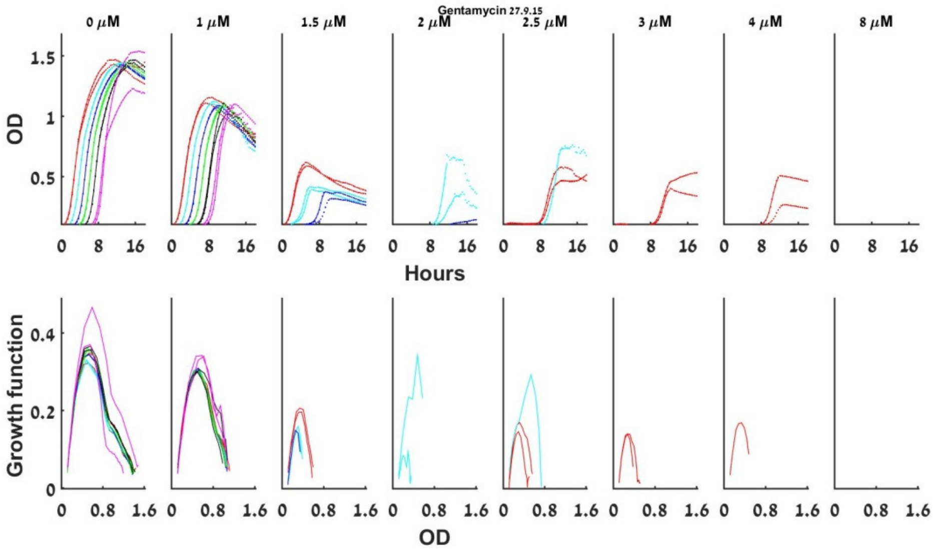

Gentamycin 27.9.15
0 μM
1 μM
1.5 μM
2 μM
2.5 μM
3 μM
4 μM
8 μM
OD
1.5
1
0.5
0
8
16
Hours
Growth function
0.4
0.2
0
0.8
1.6
OD

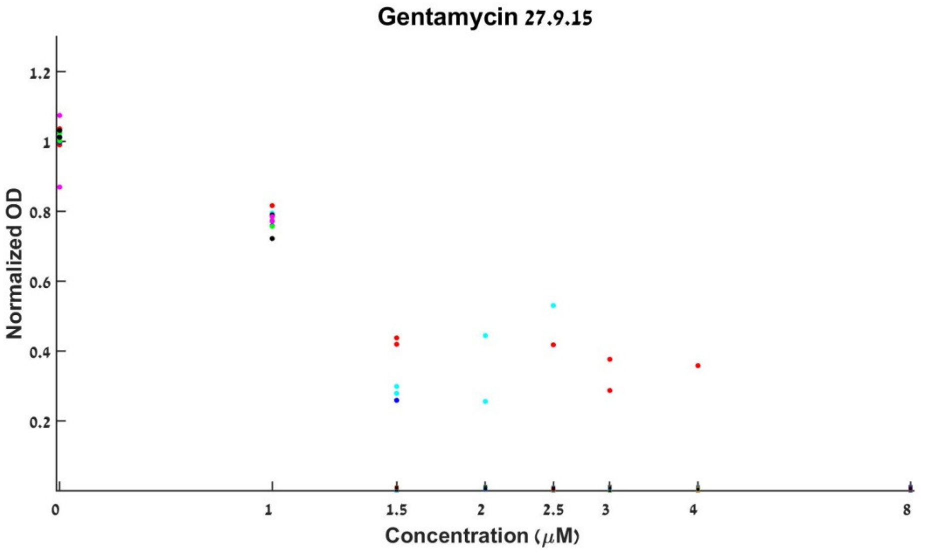

Gentamycin 27.9.15
Normalized OD
1.2
1
0.8
0.6
0.4
0.2
0
1
1.5
2
2.5
3
4
8
Concentration (μM)

Figure S5B: Kanamycin individual experiments

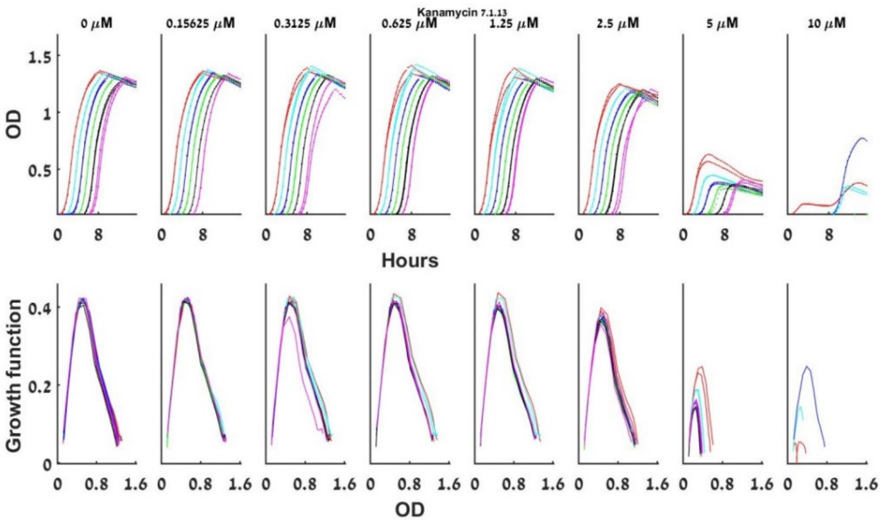

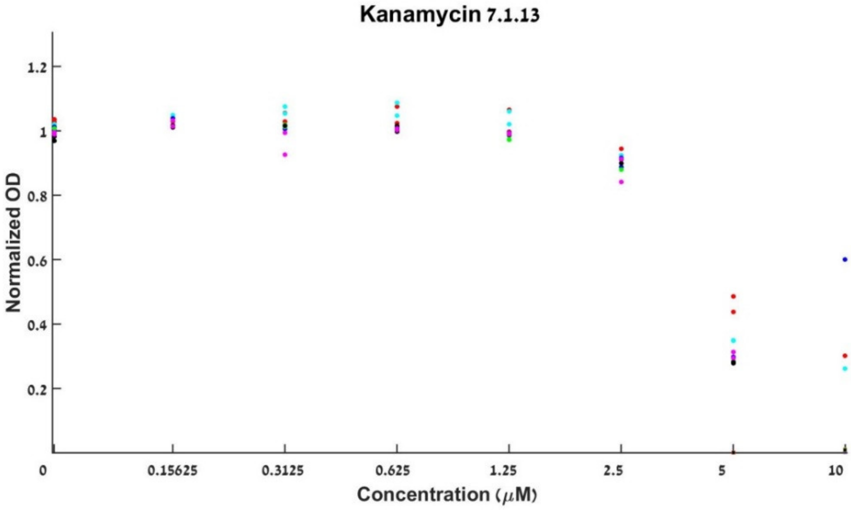

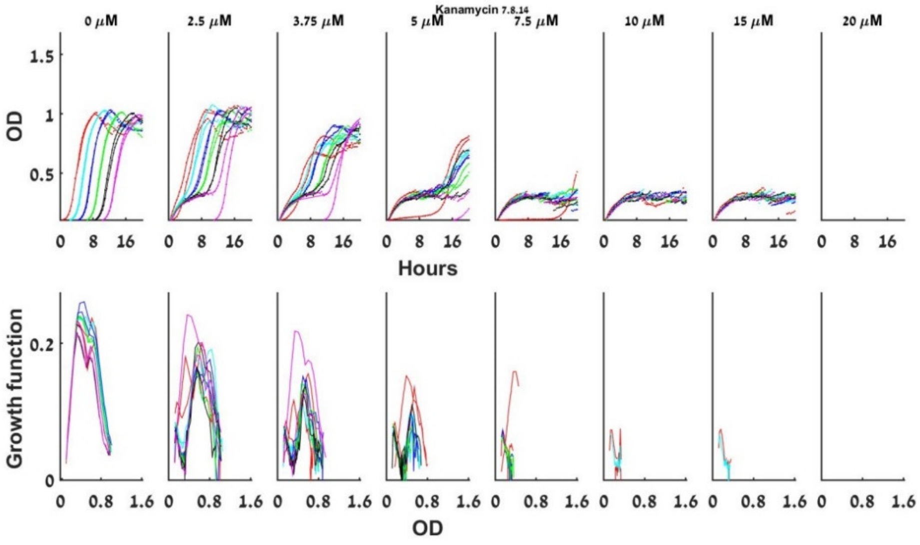

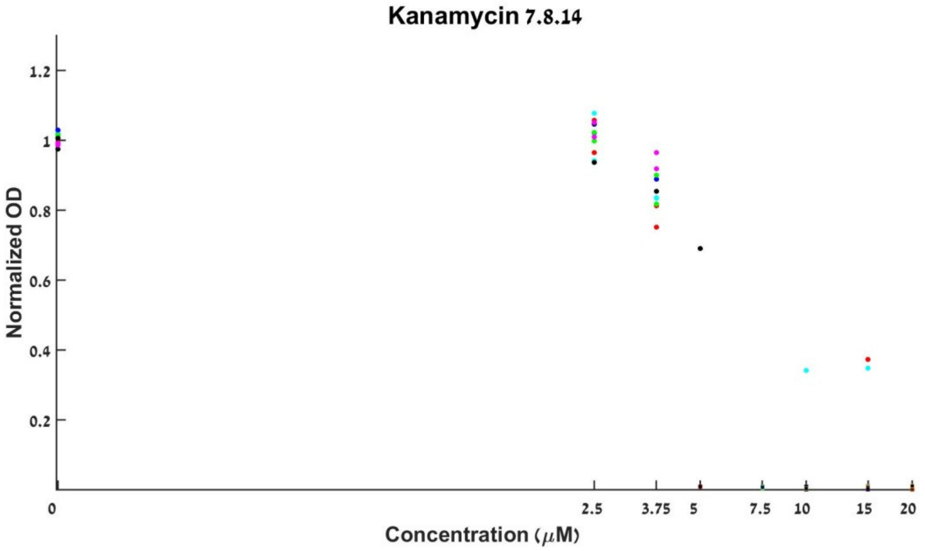
Kanamycin 7.8.14
Normalized OD
1.2
1
0.8
0.6
0.4
0.2
0
2.5
3.75
5
7.5
10
15
20
Concentration (μM)

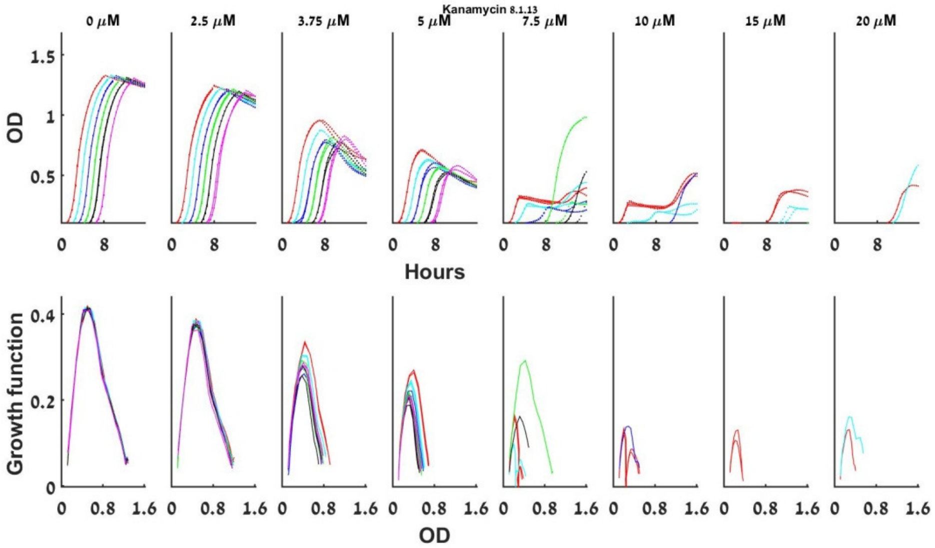
Kanamycin 8.1.13
0 μM
2.5 μM
3.75 μM
5 μM
7.5 μM
10 μM
15 μM
20 μM
OD
1.5
1
0.5
0
8
Hours
Growth function
0.4
0.2
0
0.8
1.6
OD

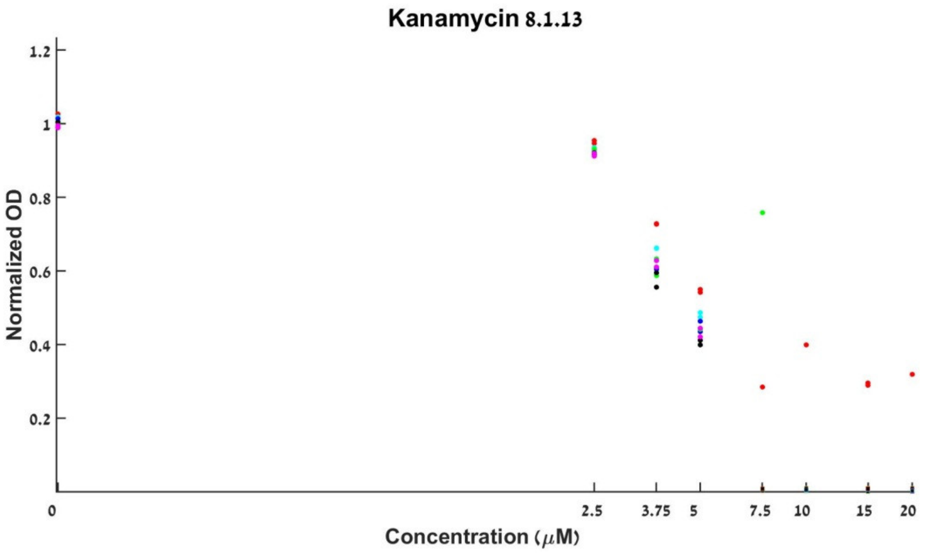
Kanamycin 8.1.13
Normalized OD
1.2
1
0.8
0.6
0.4
0.2
0
2.5
3.75
5
7.5
10
15
20
Concentration (μM)

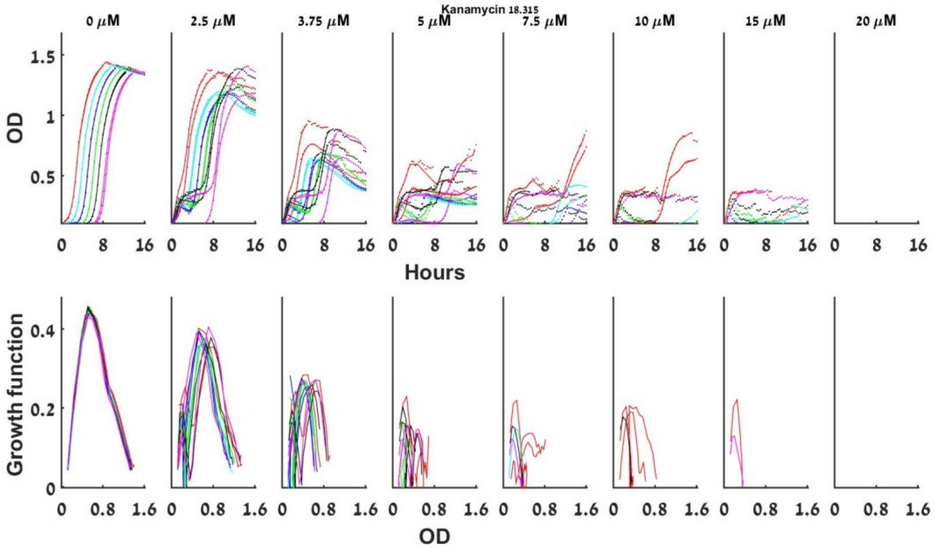
Kanamycin 18.315
0 μM
2.5 μM
3.75 μM
5 μM
7.5 μM
10 μM
15 μM
20 μM
OD
1.5
1
0.5
0
8
16
Hours
Growth function
0.4
0.2
0
0.8
1.6
OD

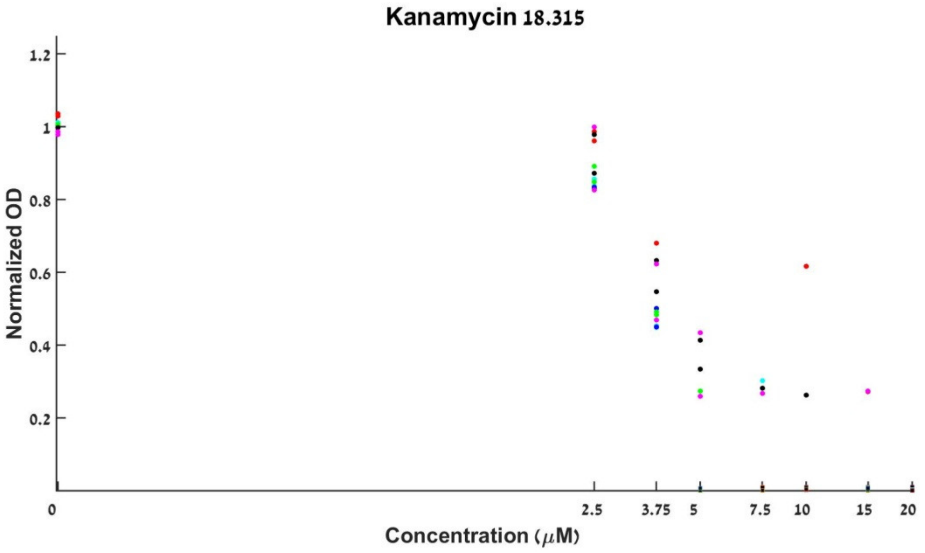
Kanamycin 18.315
Normalized OD
1.2
1
0.8
0.6
0.4
0.2
0
2.5
3.75
5
7.5
10
15
20
Concentration (μM)

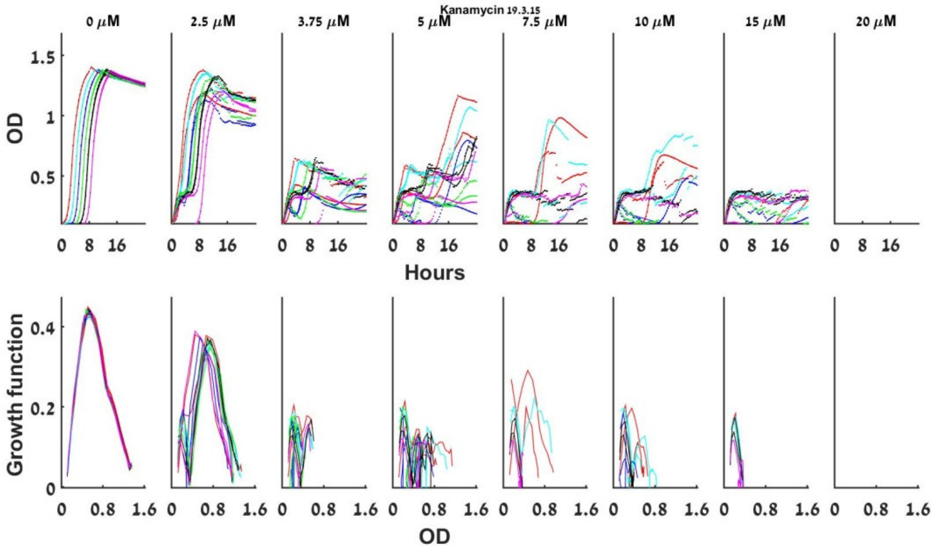
Kanamycin 19.3.15
0 μM
2.5 μM
3.75 μM
5 μM
7.5 μM
10 μM
15 μM
20 μM
OD
1.5
1
0.5
0 8 16
Hours
Growth function
0.4
0.2
0
0 0.8 1.6
OD

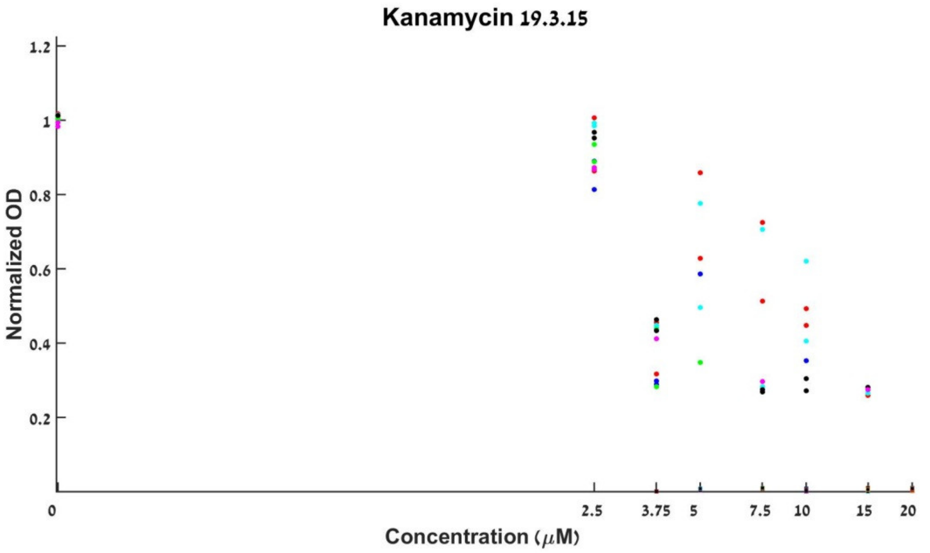
Kanamycin 19.3.15
Normalized OD
1.2
1
0.8
0.6
0.4
0.2
0
2.5
3.75
5
7.5
10
15
20
Concentration (μM)

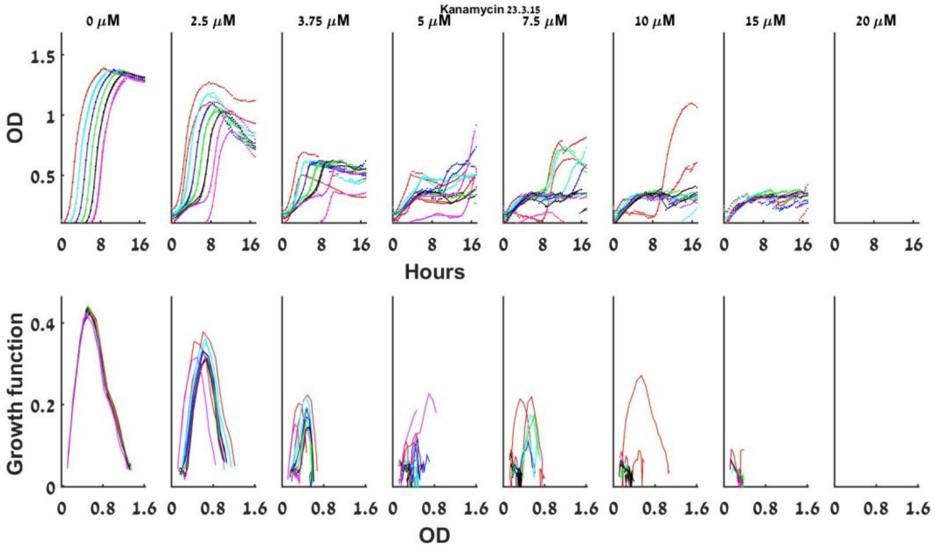
Kanamycin 23.3.15
0 μM
2.5 μM
3.75 μM
5 μM
7.5 μM
10 μM
15 μM
20 μM
OD
1.5
1
0.5
0 8 16
Hours
Growth function
0.4
0.2
0
0 0.8 1.6
OD

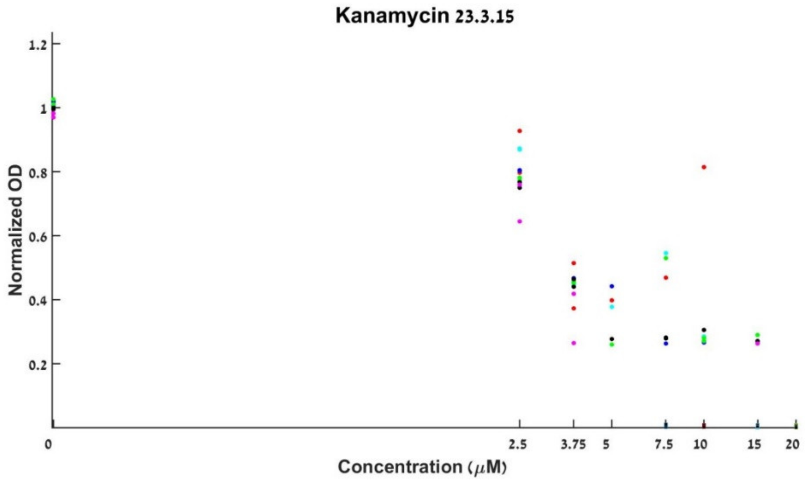

Figure S5C: Ampicillin individual experiments

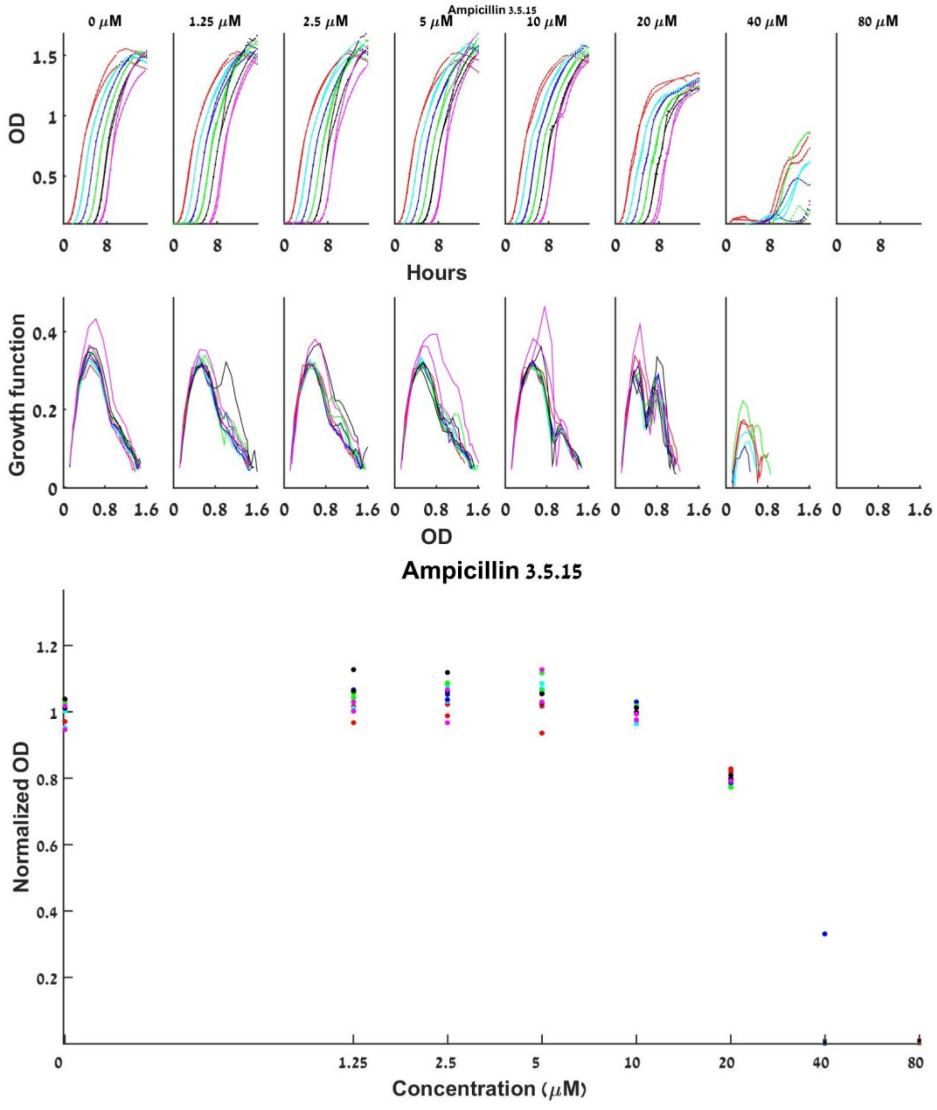

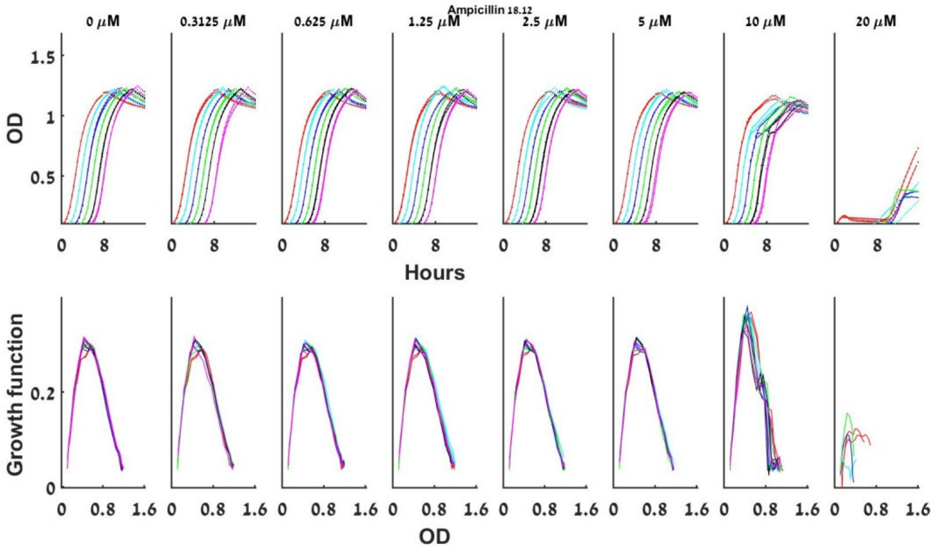

Ampicillin 18.12
0 μM
0.3125 μM
0.625 μM
1.25 μM
2.5 μM
5 μM
10 μM
20 μM
OD
Hours
Growth function
OD

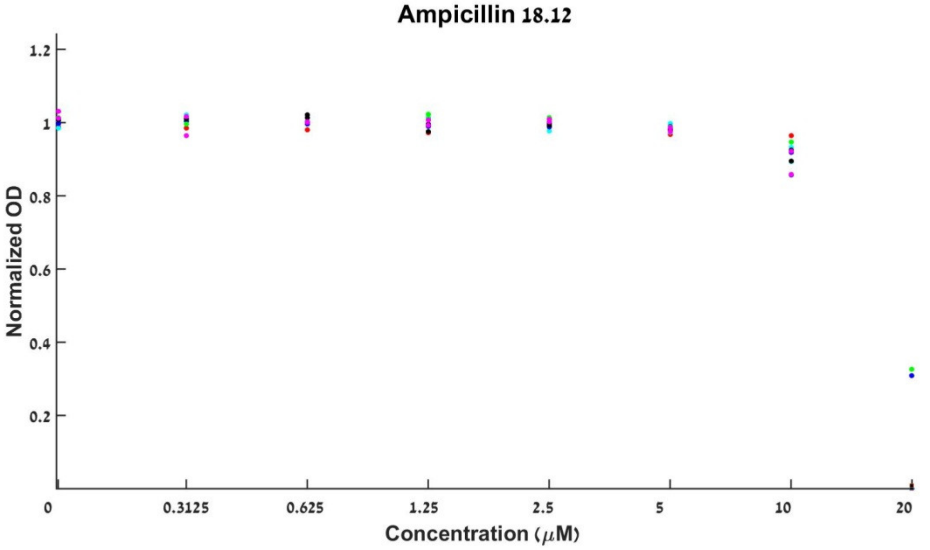

Ampicillin 18.12
Normalized OD
Concentration (μM)
0
0.3125
0.625
1.25
2.5
5
10
20

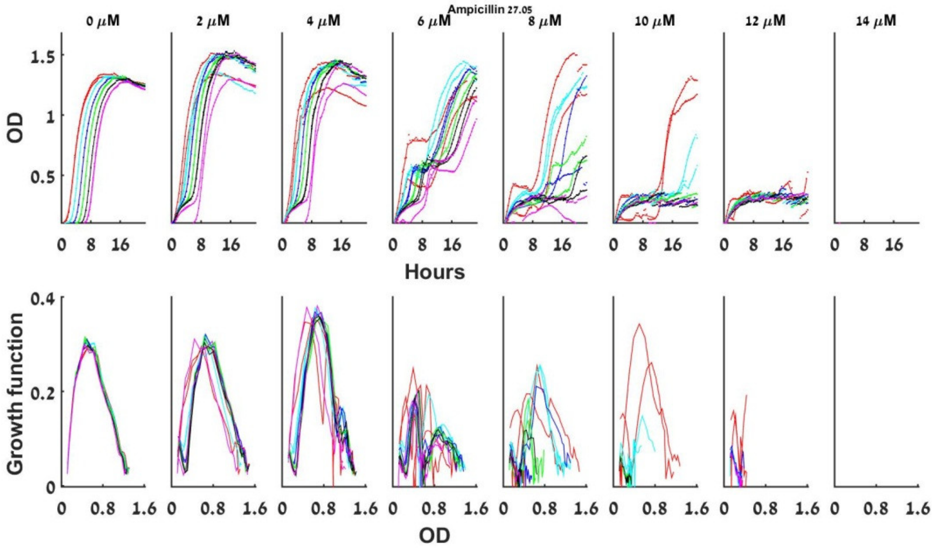

Ampicillin 27.05
0 μM
2 μM
4 μM
6 μM
8 μM
10 μM
12 μM
14 μM
OD
Hours
Growth function
OD

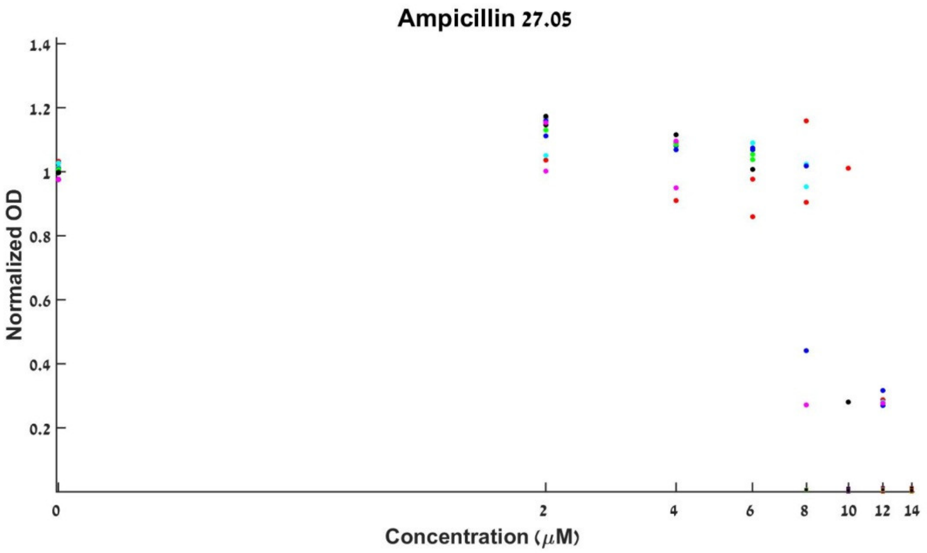
Ampicillin 27.05
Normalized OD
Concentration (μM)

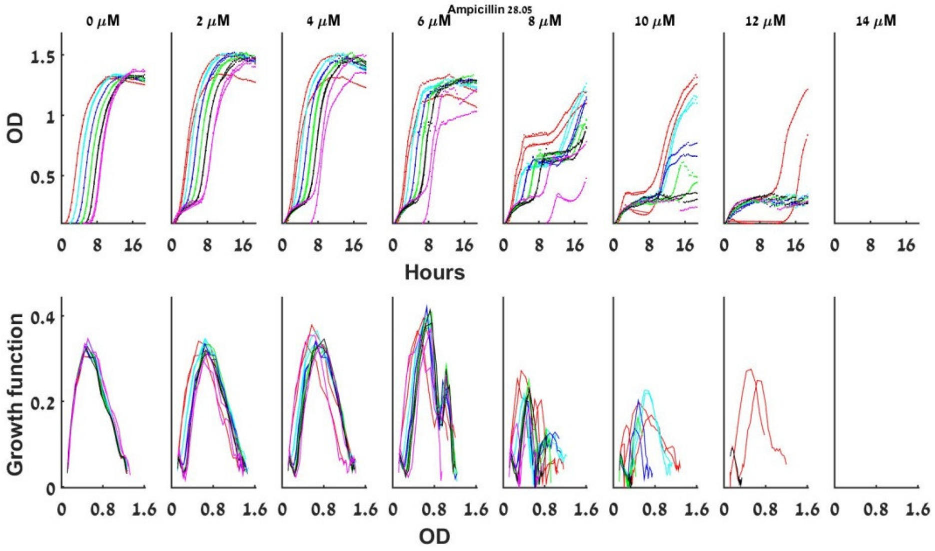
Ampicillin 28.05
0 μM
2 μM
4 μM
6 μM
8 μM
10 μM
12 μM
14 μM
OD
Hours
Growth function
OD

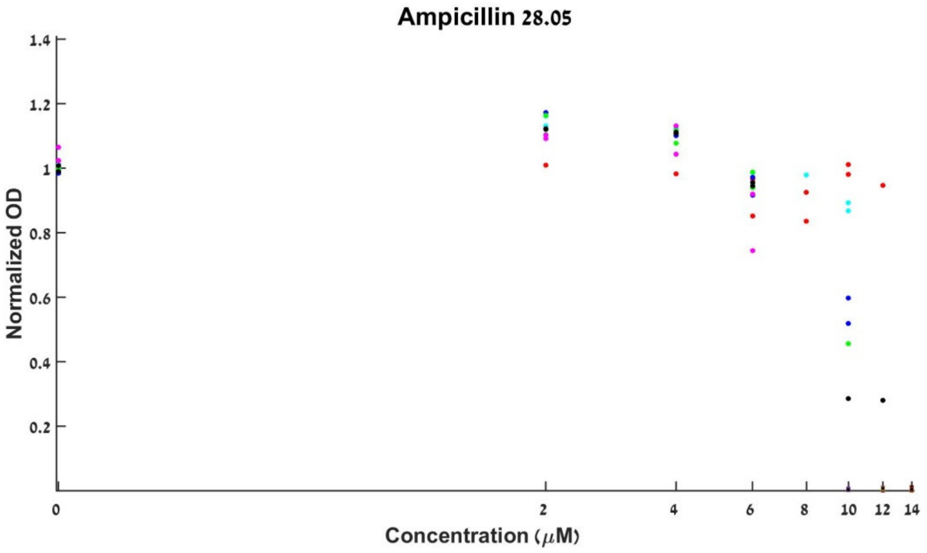
Ampicillin 28.05
Normalized OD
Concentration (μM)

Figure S5D: Carbenicllin individual experiments

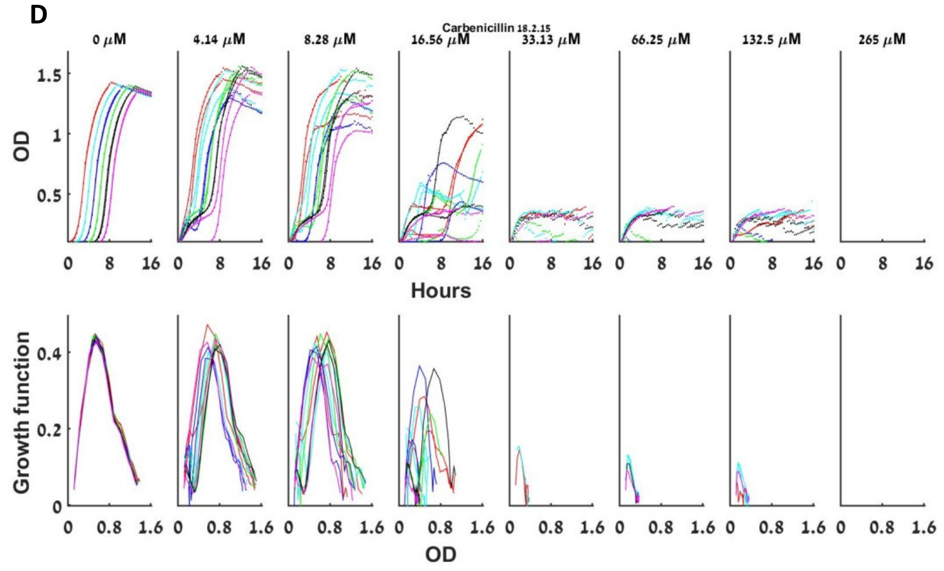

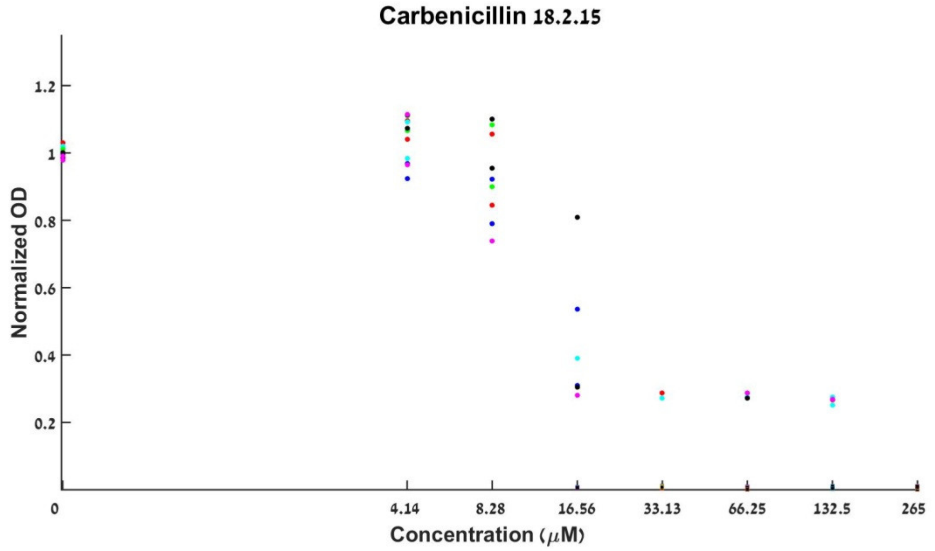

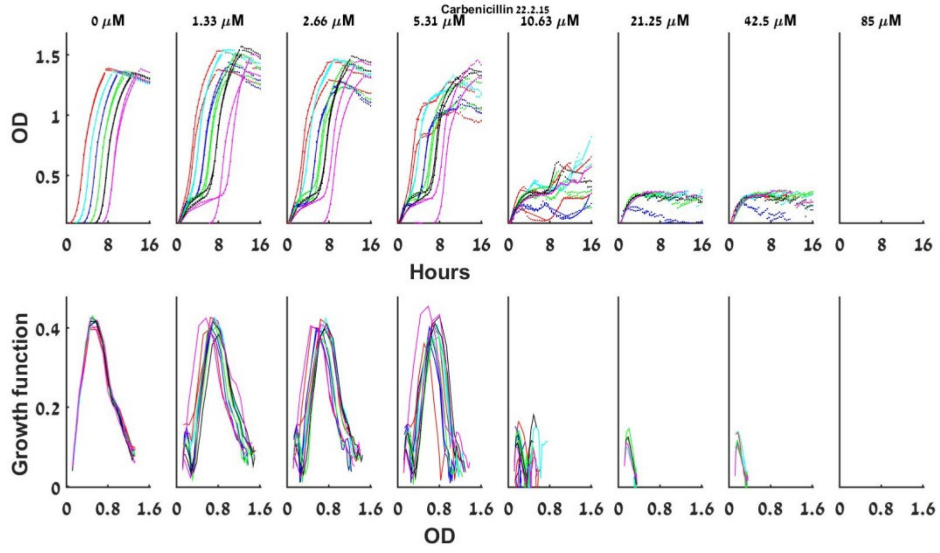

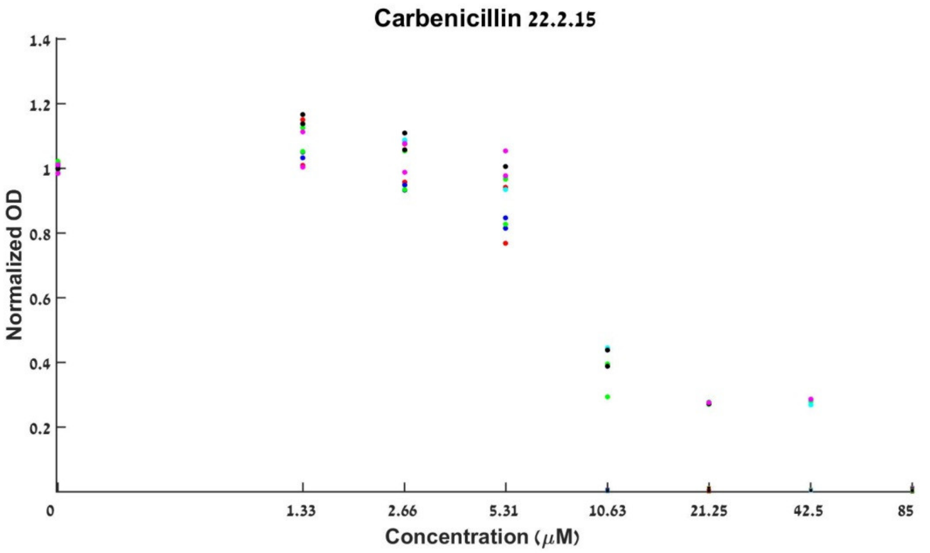
Carbenicillin 22.2.15
Normalized OD
1.4
1.2
1
0.8
0.6
0.4
0.2
0
1.33
2.66
5.31
10.63
21.25
42.5
85
Concentration (μM)

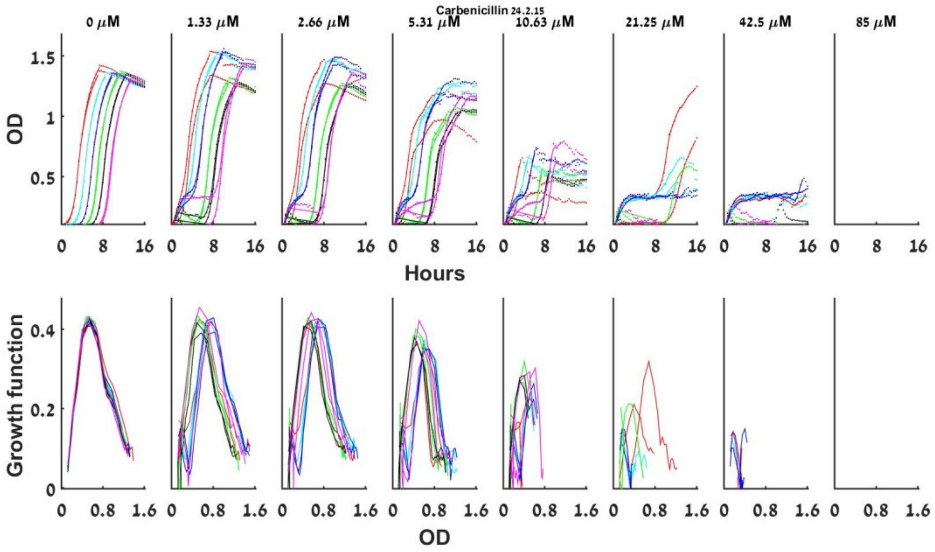
Carbenicillin 24.2.15
0 μM
1.33 μM
2.66 μM
5.31 μM
10.63 μM
21.25 μM
42.5 μM
85 μM
OD
1.5
1
0.5
0 8 16
Hours
Growth function
0.4
0.2
0
0 0.8 1.6
OD

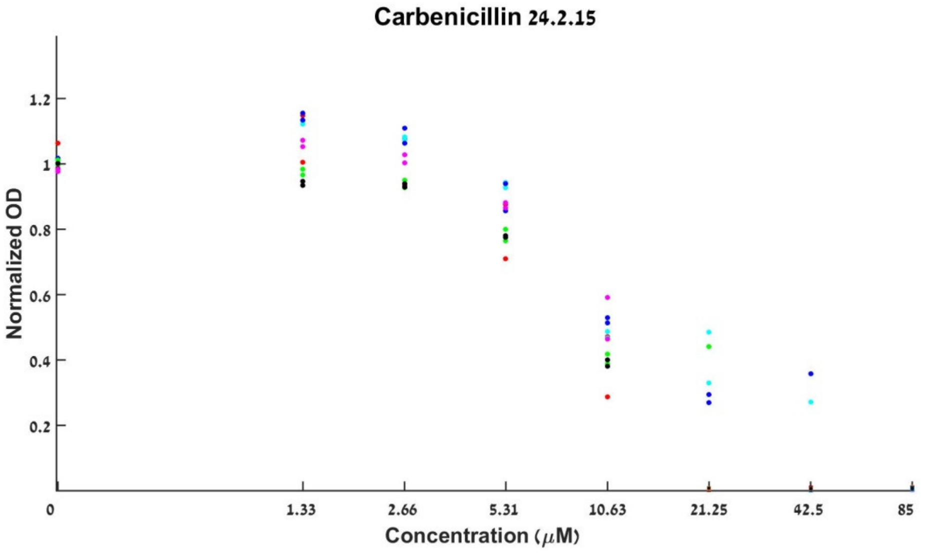
Carbenicillin 24.2.15
Normalized OD
1.2
1
0.8
0.6
0.4
0.2
0
1.33
2.66
5.31
10.63
21.25
42.5
85
Concentration (μM)

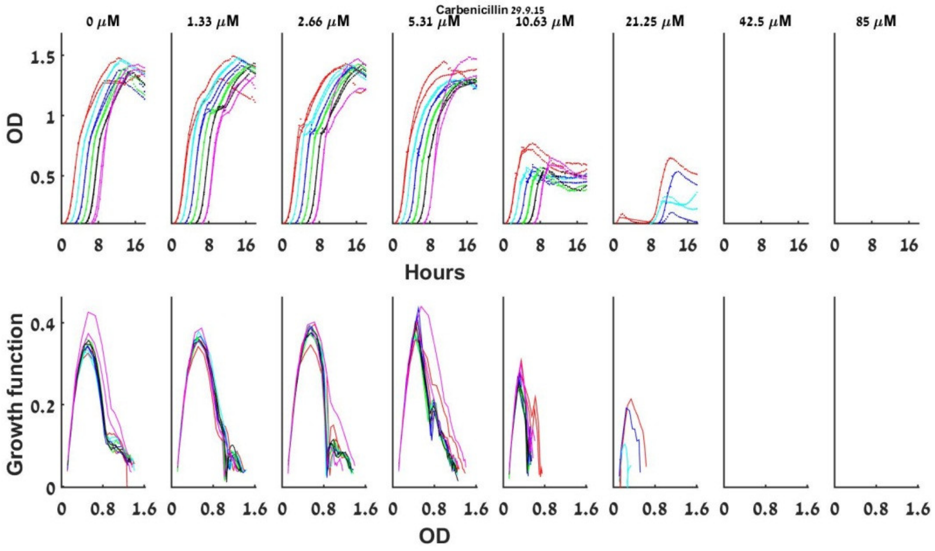

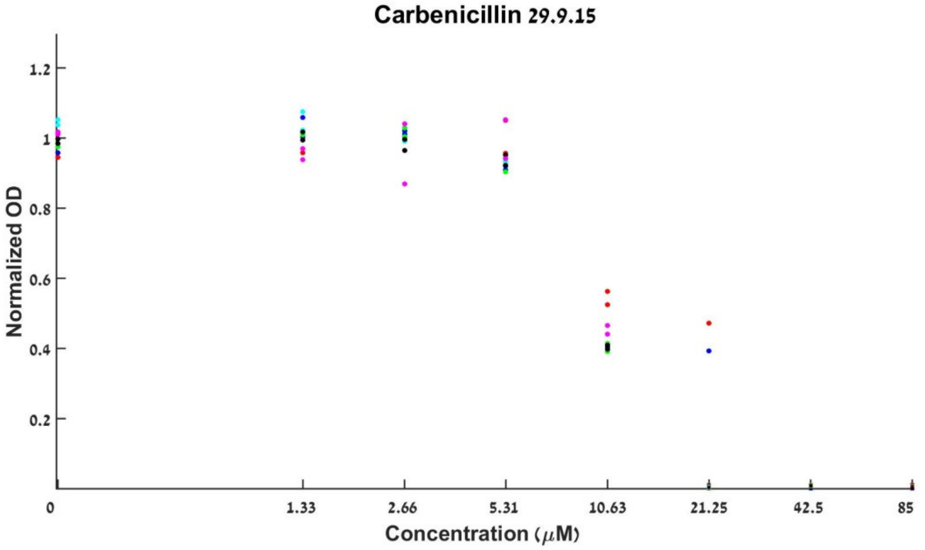

Figure S5. Bacteria grown with β-lactam or aminoglycoside antibiotics exhibit BMFD. Growth curves, growth functions and maximal capacity of single experiments performed with β-lactam or aminoglycoside antibiotics (**A**) Single IE experiments with Gentamycin. (**B**) Single IE experiments with Kanamycin. (**C**) Single IE experiments with Ampicillin. (**D**) Single IE experiments with Carbenicillin.

β -lactamase resistant β-lactams induce different dynamics than regular β-lactams dynamics

Growth curves, growth functions and maximal capacity of all experiments performed with Oxacillin (Figure S6).

Figure S6

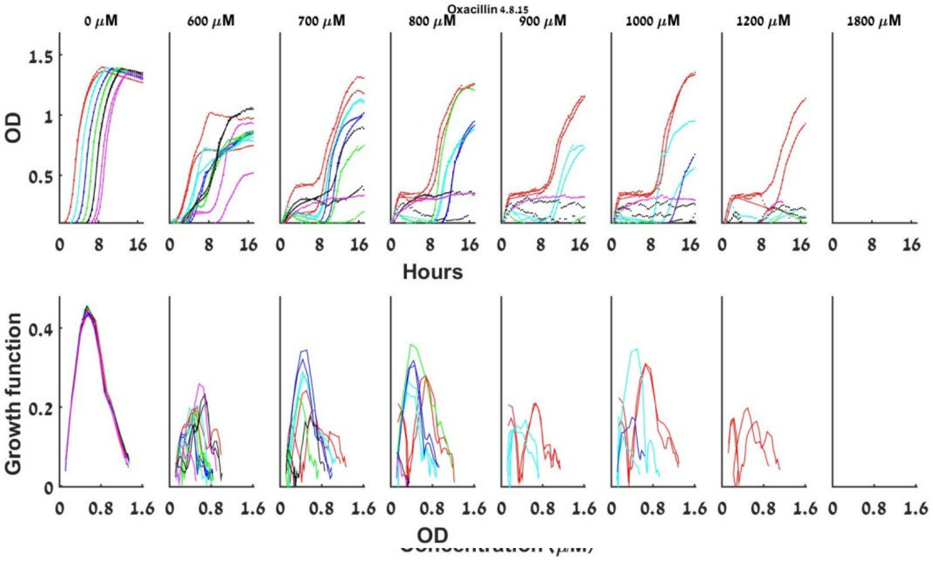

Oxacillin 4.8.15
0 μM
600 μM
700 μM
800 μM
900 μM
1000 μM
1200 μM
1800 μM
OD
Hours
Growth function
OD

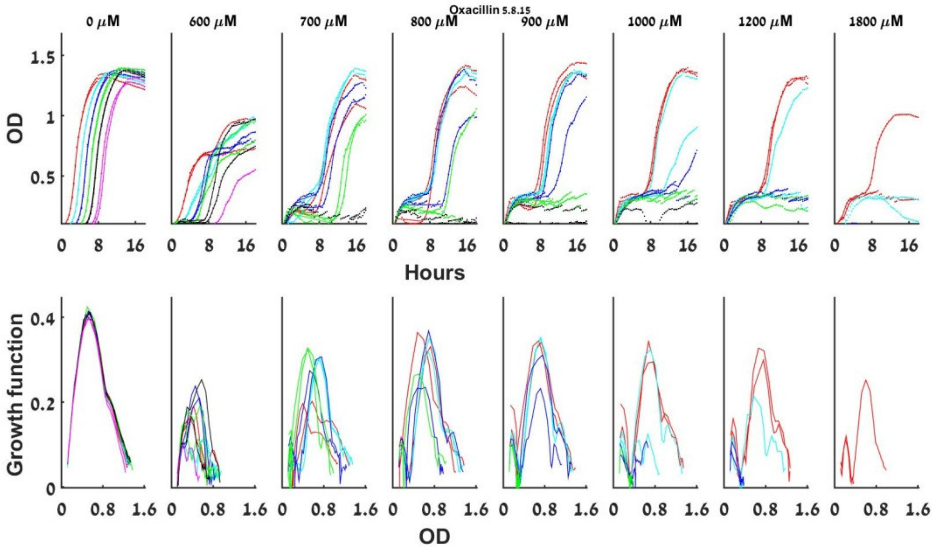

Oxacillin 5.8.15
0 μM
600 μM
700 μM
800 μM
900 μM
1000 μM
1200 μM
1800 μM
OD
Hours
Growth function
OD

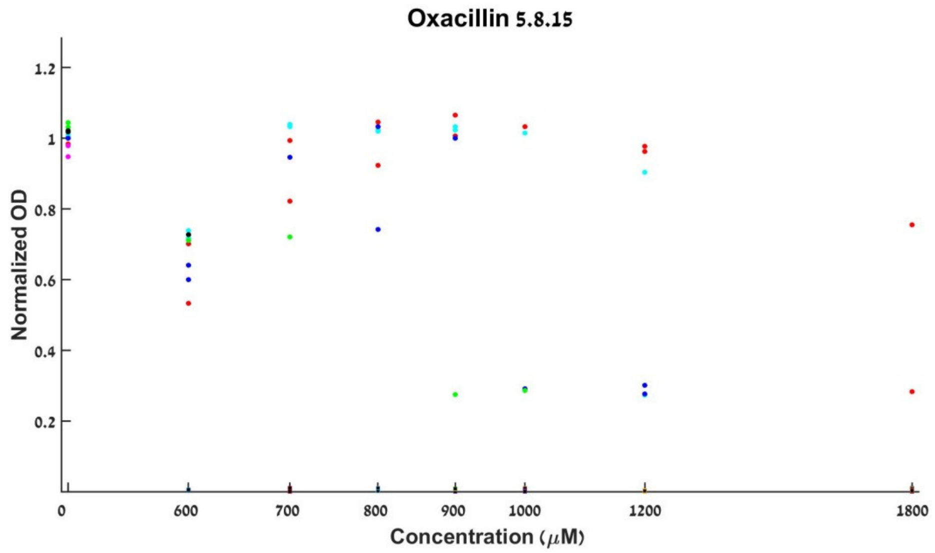
Oxacillin 5.8.15
Normalized OD
Concentration (μM)
0
600
700
800
900
1000
1200
1800

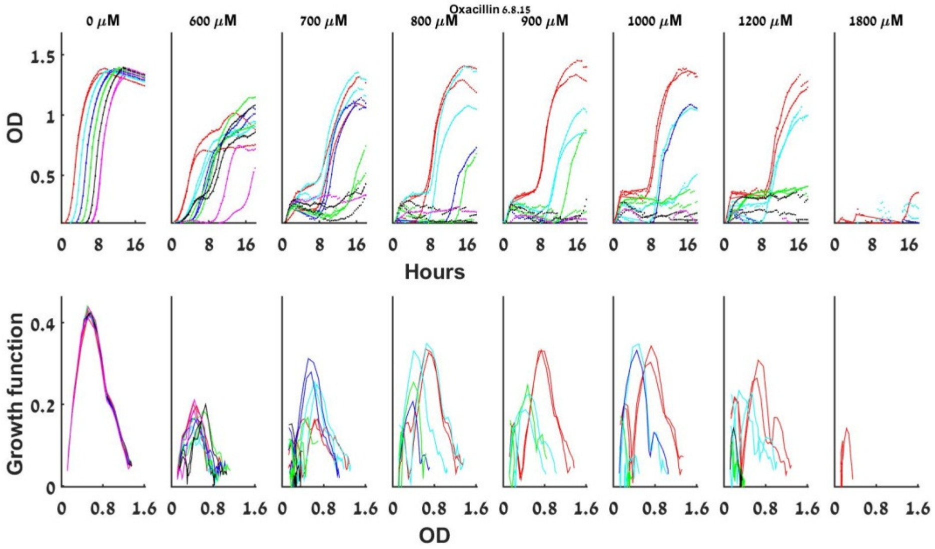
Oxacillin 6.8.15
0 μM
600 μM
700 μM
800 μM
900 μM
1000 μM
1200 μM
1800 μM
OD
Hours
Growth function
OD

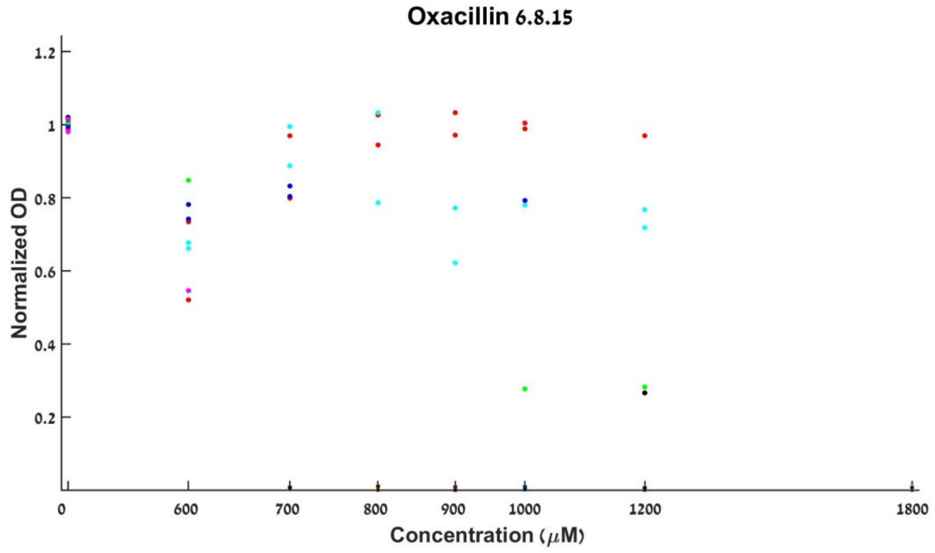
Oxacillin 6.8.15
Normalized OD
Concentration (μM)
0
600
700
800
900
1000
1200
1800

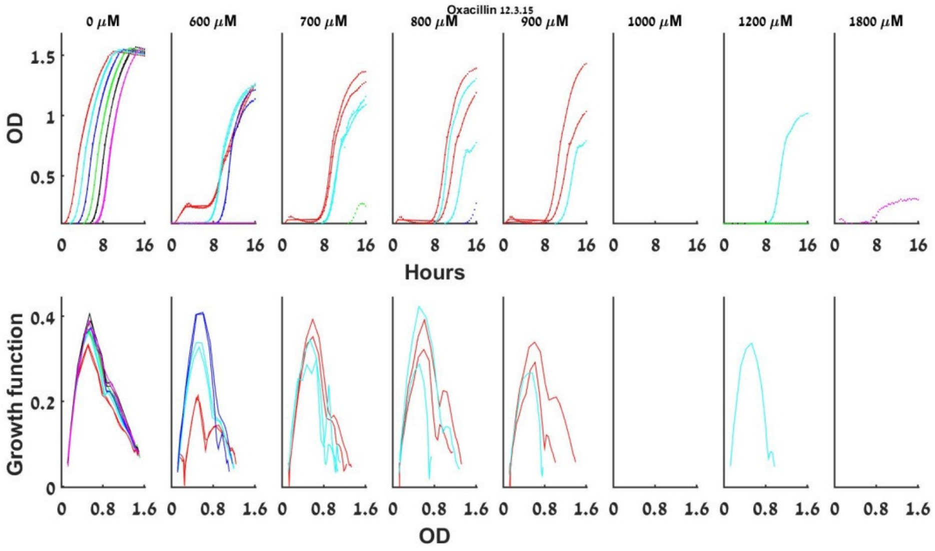
Oxacillin 12.3.15
0 μM
600 μM
700 μM
800 μM
900 μM
1000 μM
1200 μM
1800 μM
OD
Hours
Growth function
OD

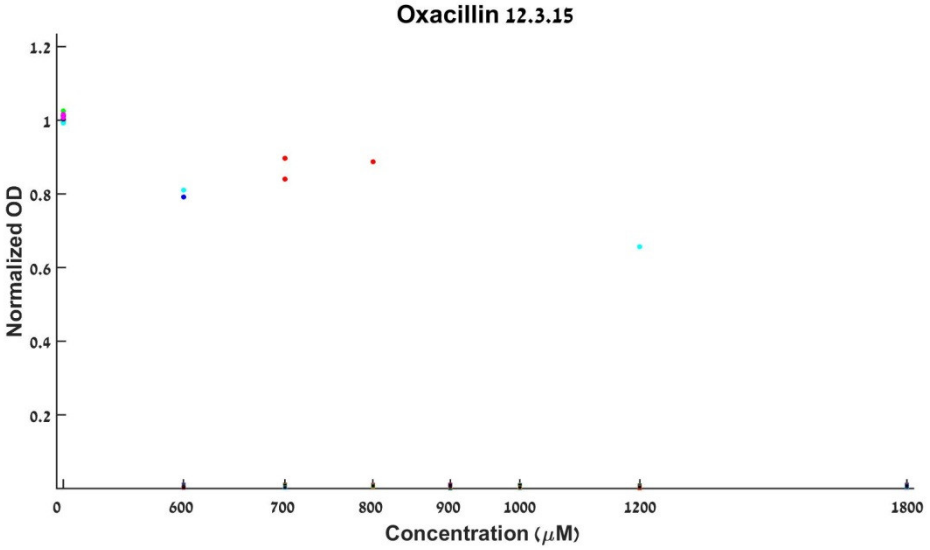
Oxacillin 12.3.15
Normalized OD
Concentration (μM)

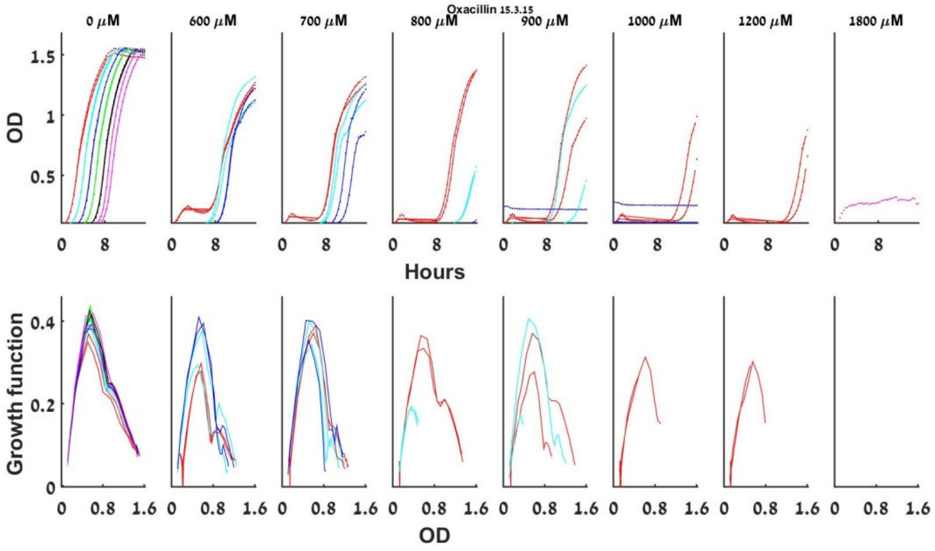
Oxacillin 15.3.15
0 μM
600 μM
700 μM
800 μM
900 μM
1000 μM
1200 μM
1800 μM
OD
Hours
Growth function
OD

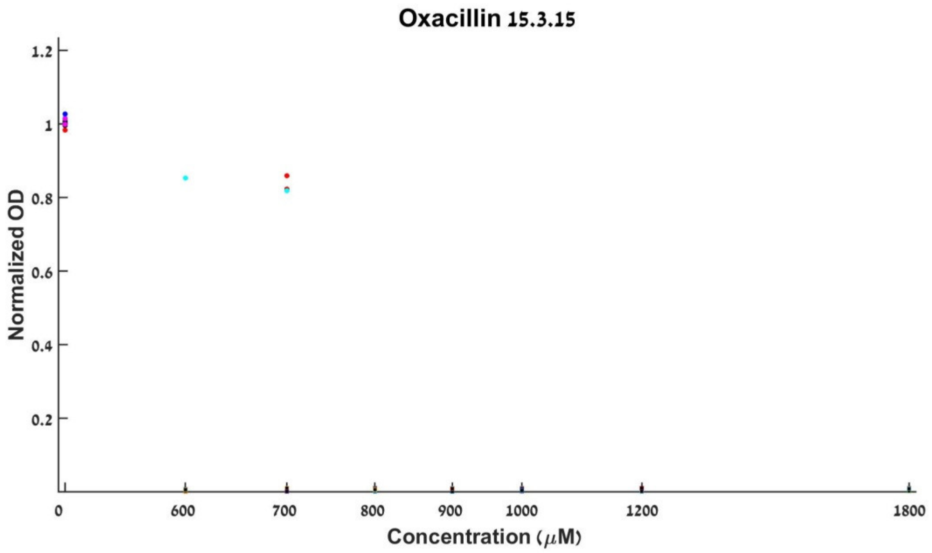
Oxacillin 15.3.15
Normalized OD
Concentration (μM)
0
600
700
800
900
1000
1200
1800

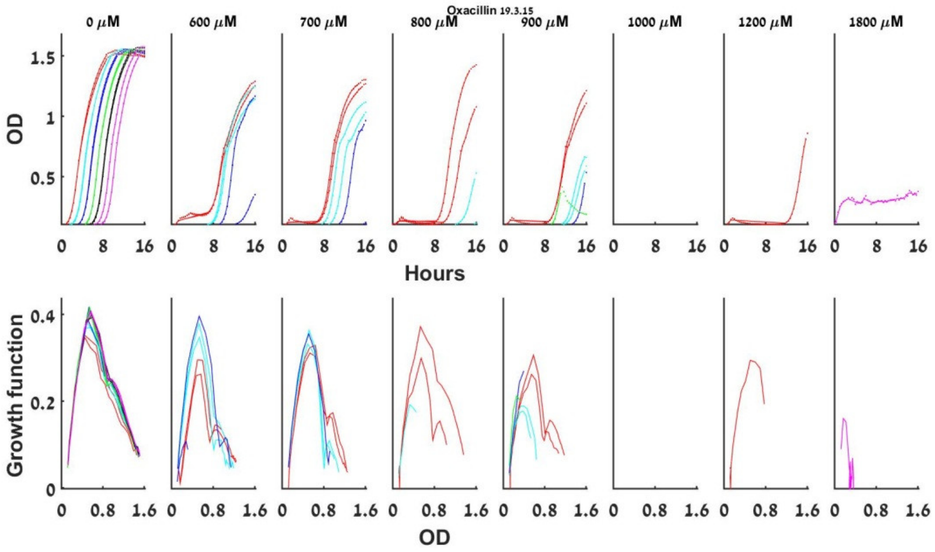
Oxacillin 19.3.15
0 μM
600 μM
700 μM
800 μM
900 μM
1000 μM
1200 μM
1800 μM
OD
Hours
Growth function
OD

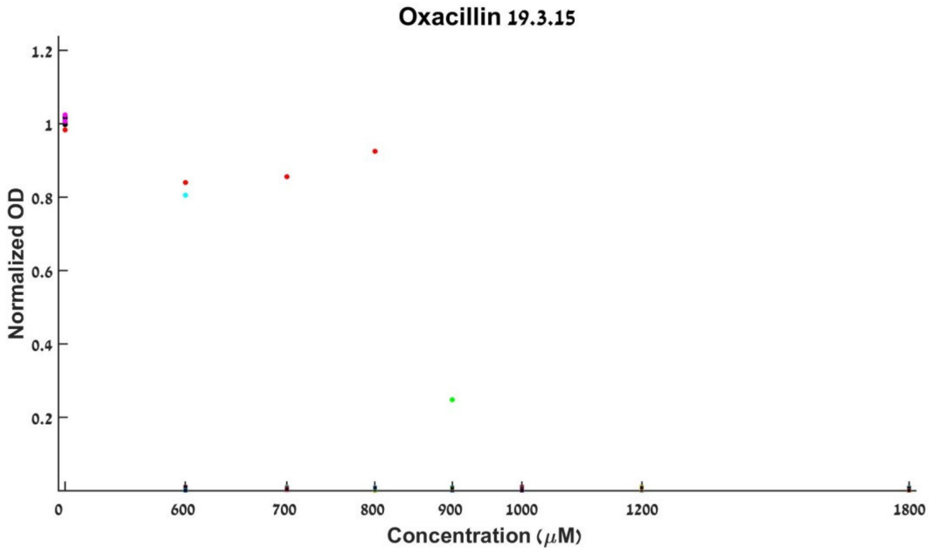
Oxacillin 19.3.15
Normalized OD
Concentration (μM)
0
600
700
800
900
1000
1200
1800

Figure S6. Bacterial growth dynamics with Oxacillin exhibit BMFD which is antibiotic concentration independent. Growth curves, growth functions and maximal capacity of single experiments performed with Oxacillin.

E.coli K12 grown in the presence of antimicrobial peptides exhibit bistable kill and A-independent dynamics (BIK)

Growth curves, growth functions and maximal capacity of all experiments performed with antimicrobial peptides - Polymixin B, MSI, Melittin and K6L9 (Figures S7A-D), maximal capacity of 6 experiments with Melittin together or 4 experiments with K6L9 (Figure S7E).

Figures S7A: Polymixin B individual experiments

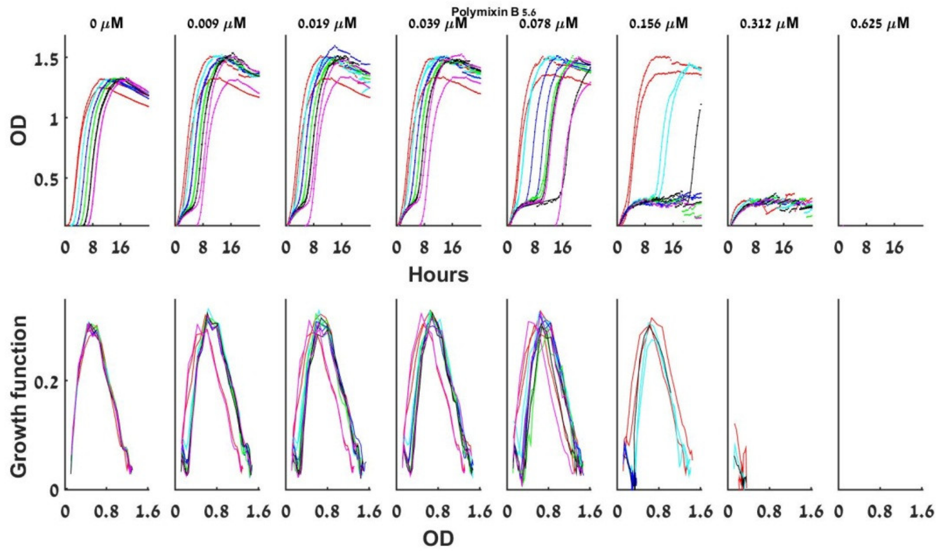

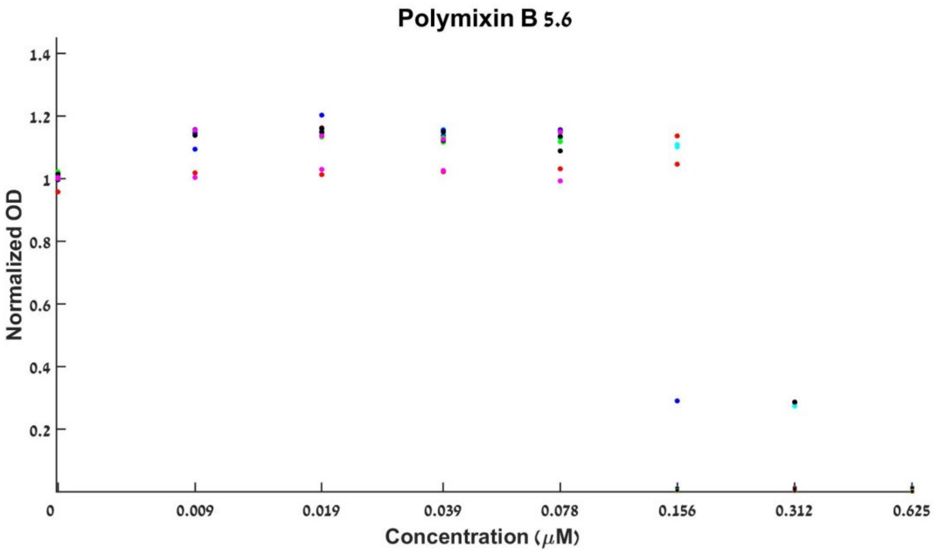
Polymixin B 5.6
Normalized OD
1.4
1.2
1
0.8
0.6
0.4
0.2
0
0.009
0.019
0.039
0.078
0.156
0.312
0.625
Concentration (μM)

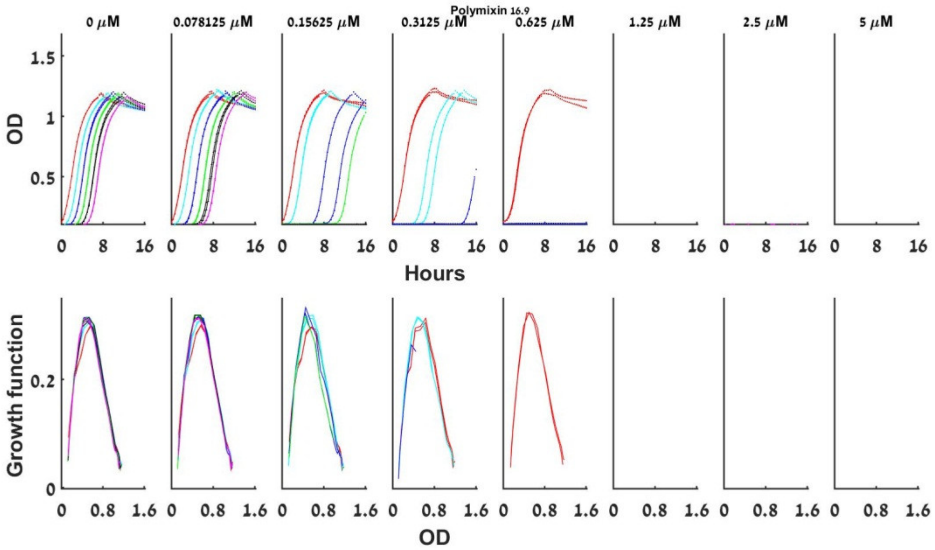
Polymixin 16.9
0 μM
0.078125 μM
0.15625 μM
0.3125 μM
0.625 μM
1.25 μM
2.5 μM
5 μM
OD
1.5
1
0.5
0 8 16
Hours
Growth function
0.2
0
0 0.8 1.6
OD

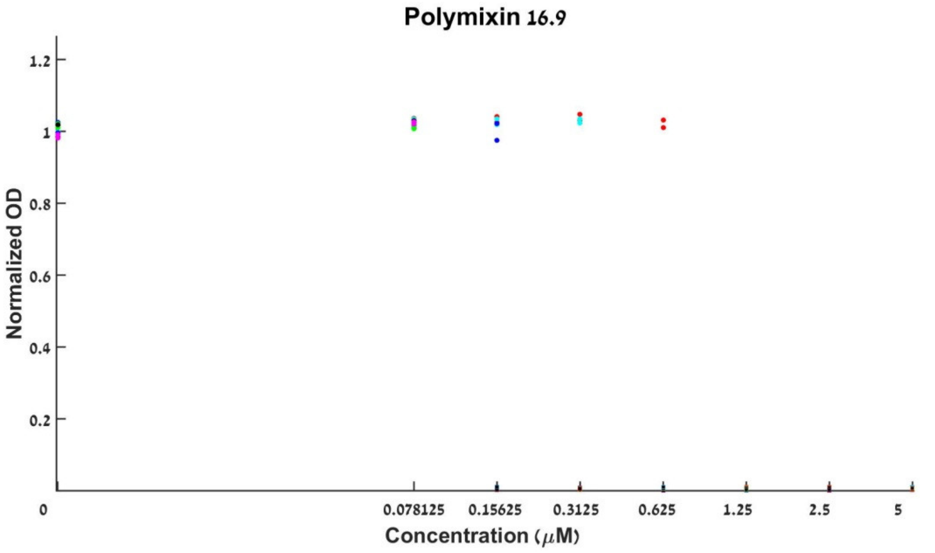
Polymixin 16.9
Normalized OD
1.2
1
0.8
0.6
0.4
0.2
0
0.078125
0.15625
0.3125
0.625
1.25
2.5
5
Concentration (μM)

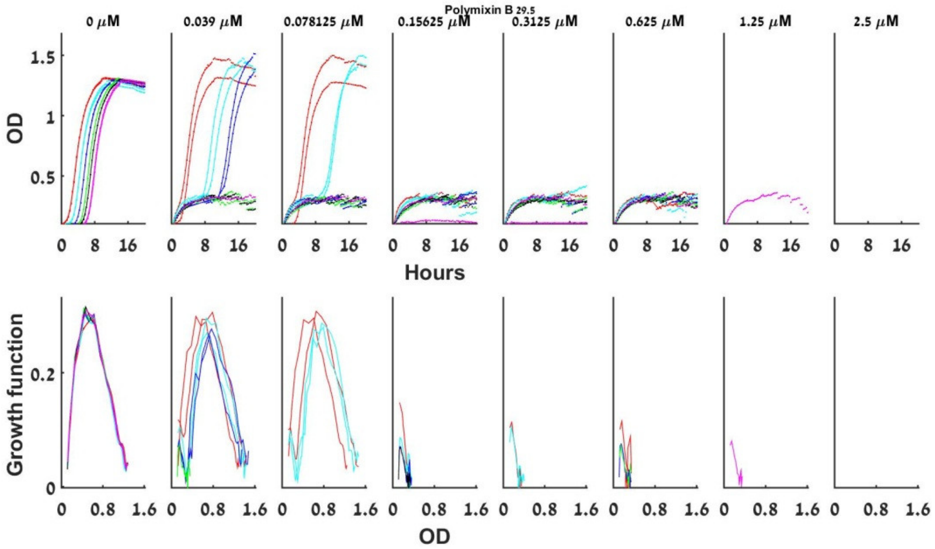

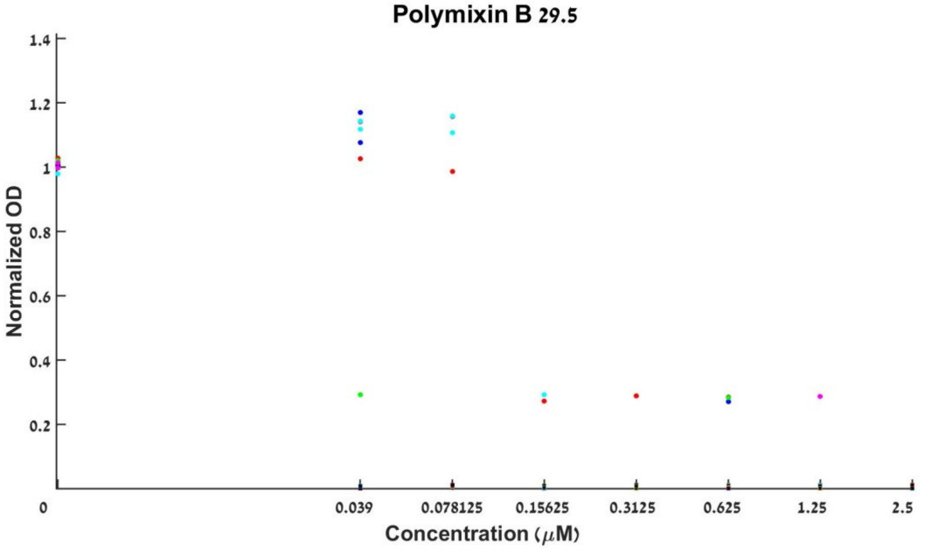

Figures S7B: MSI individual experiments

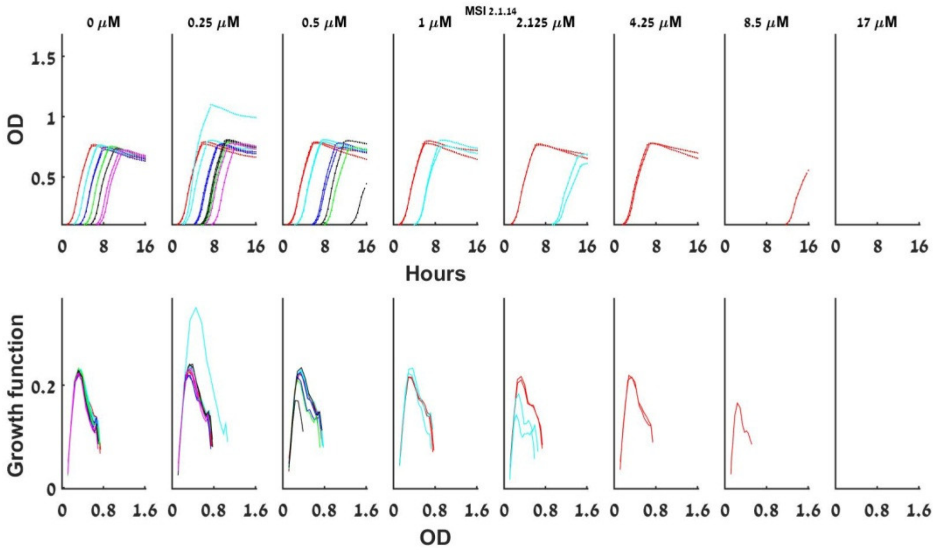

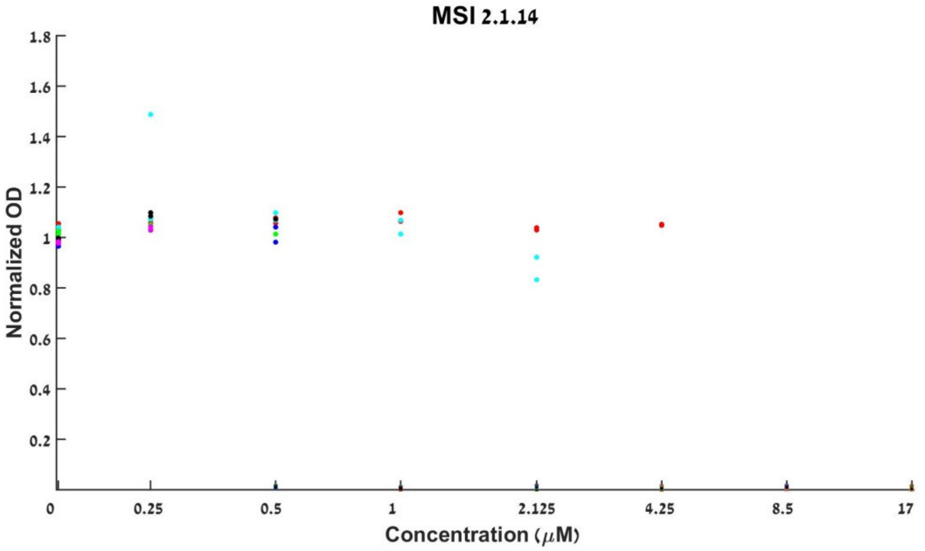
MSI 2.1.14
Normalized OD
Concentration (μM)
0
0.25
0.5
1
2.125
4.25
8.5
17

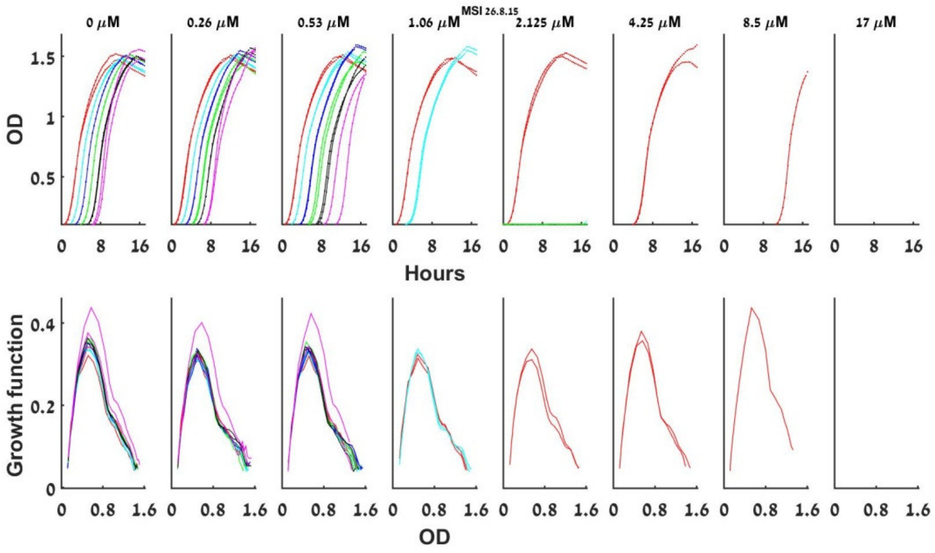
MSI 26.8.15
0 μM
0.26 μM
0.53 μM
1.06 μM
2.125 μM
4.25 μM
8.5 μM
17 μM
OD
Hours
Growth function
OD

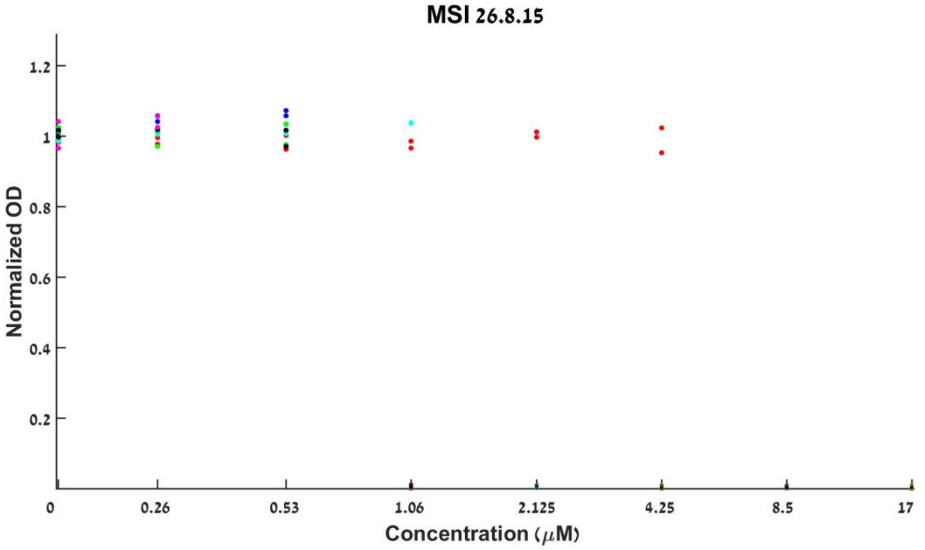
MSI 26.8.15
Normalized OD
Concentration (μM)
0
0.26
0.53
1.06
2.125
4.25
8.5
17

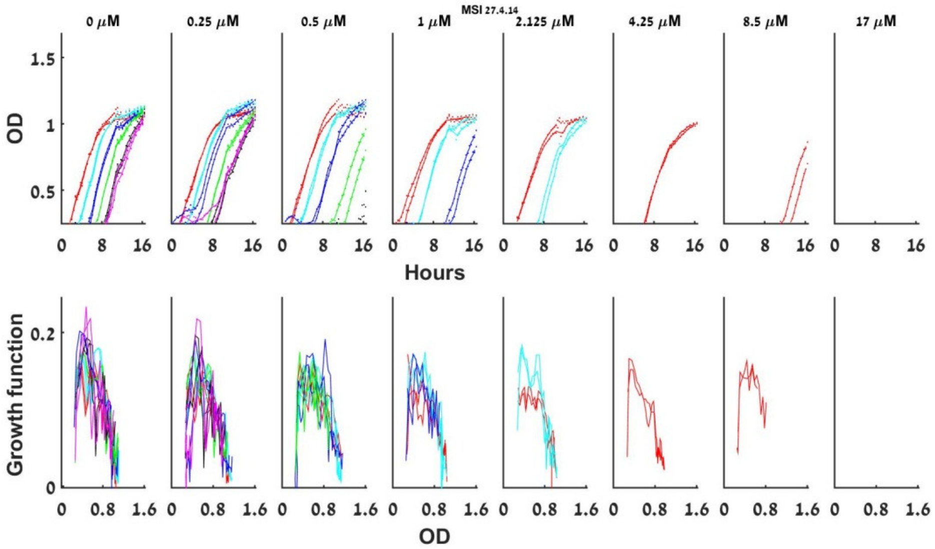
MSI 27.4.14
0 μM
0.25 μM
0.5 μM
1 μM
2.125 μM
4.25 μM
8.5 μM
17 μM
OD
Hours
Growth function
OD

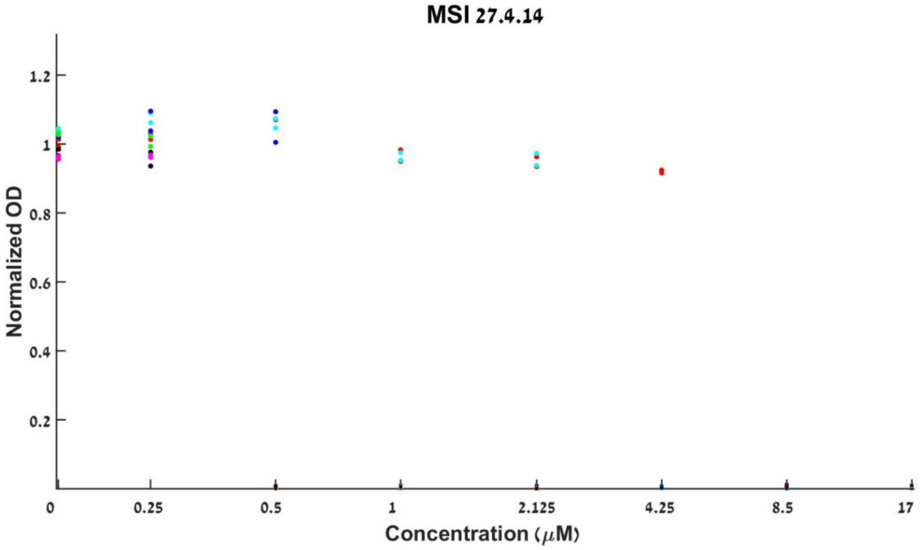
MSI 27.4.14
Normalized OD
Concentration (μM)
0
0.25
0.5
1
2.125
4.25
8.5
17

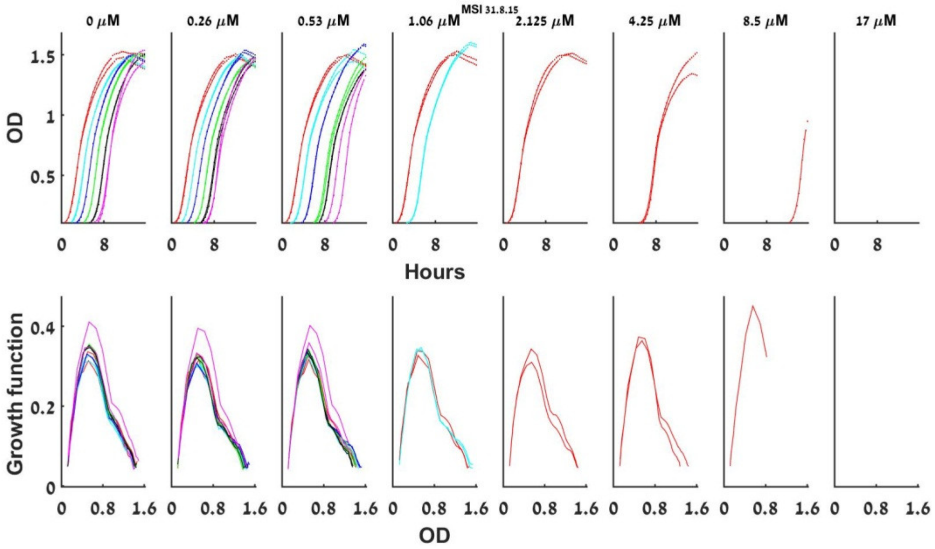
MSI 31.8.15
0 μM
0.26 μM
0.53 μM
1.06 μM
2.125 μM
4.25 μM
8.5 μM
17 μM
OD
Hours
Growth function
OD

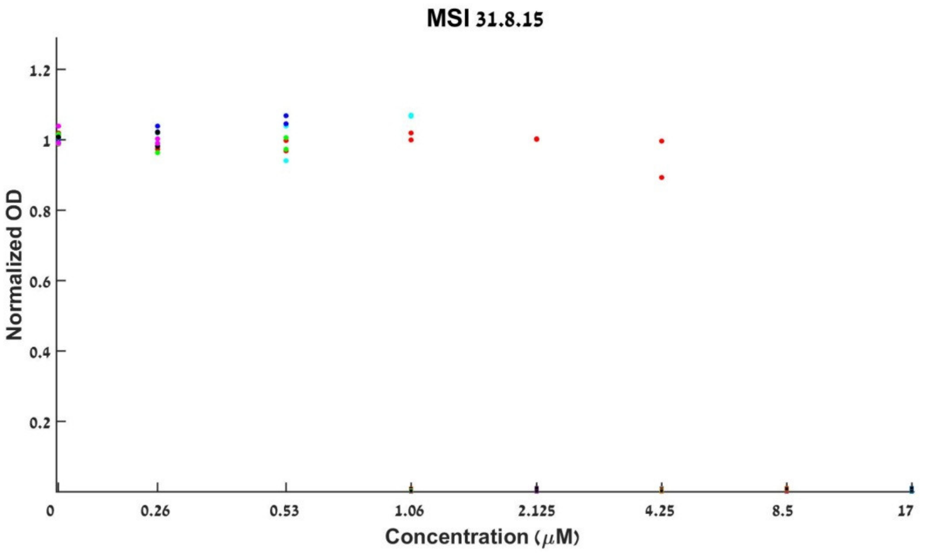

Figures S7C: Melittin individual experiments

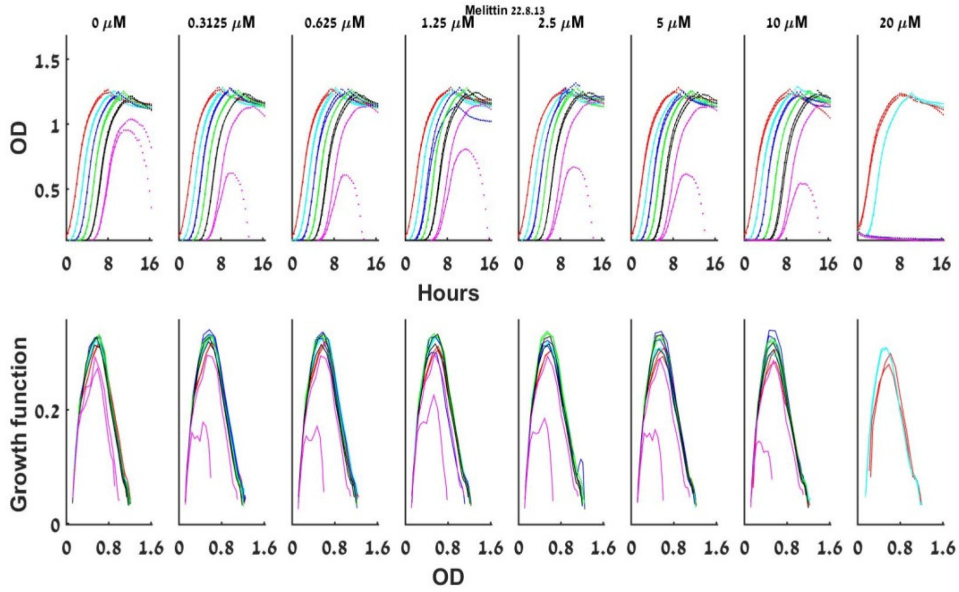

Melittin 22.8.13

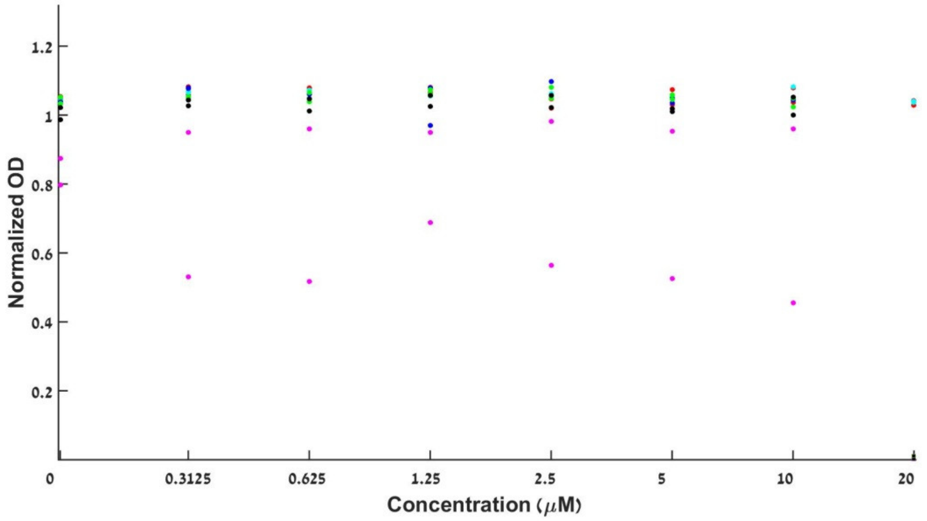

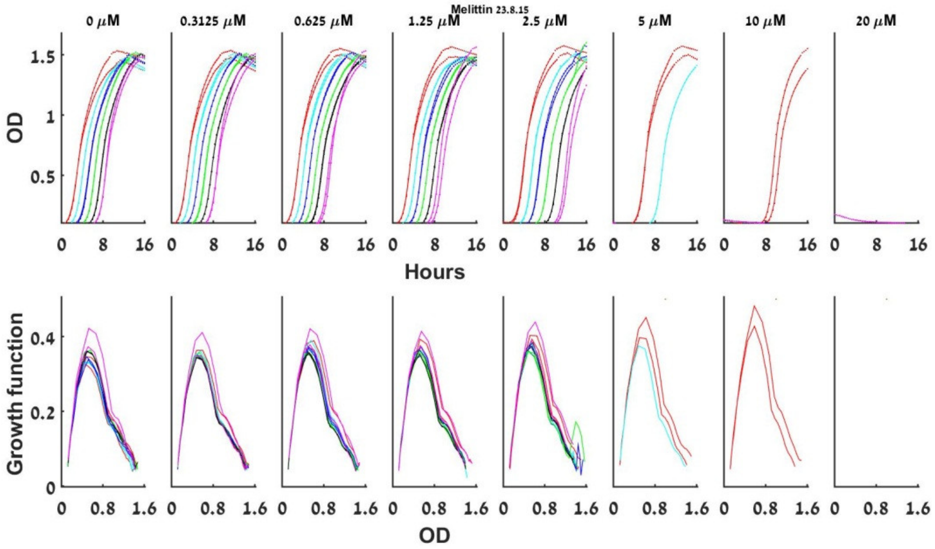

Melittin 23.8.15

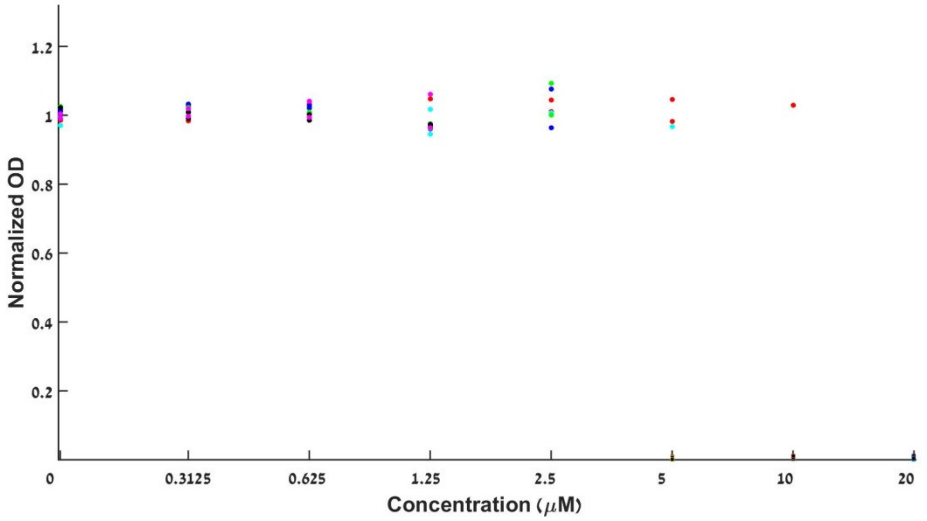

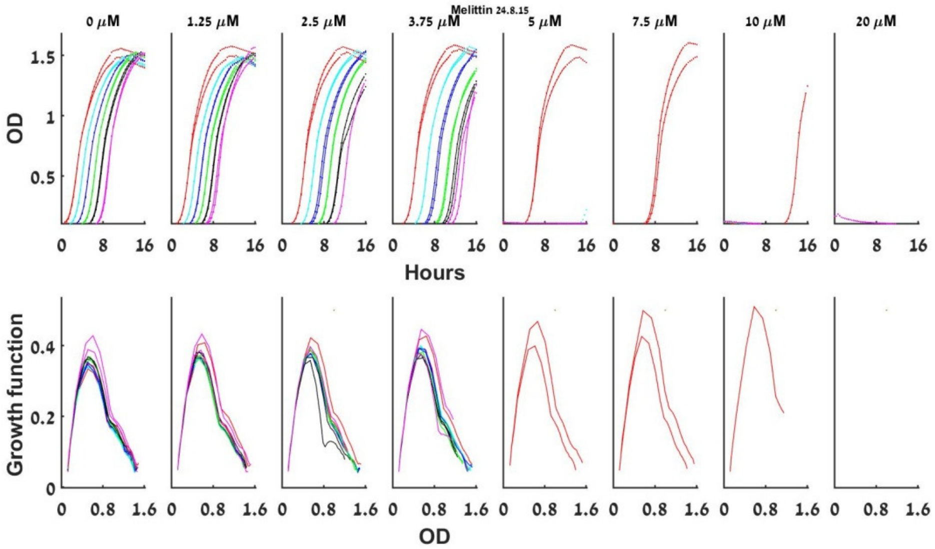
Melittin 24.8.15
0 μM
1.25 μM
2.5 μM
3.75 μM
5 μM
7.5 μM
10 μM
20 μM
OD
0.5
1
1.5
0
8
16
Hours
Growth function
0
0.2
0.4
0.8
1.6
OD

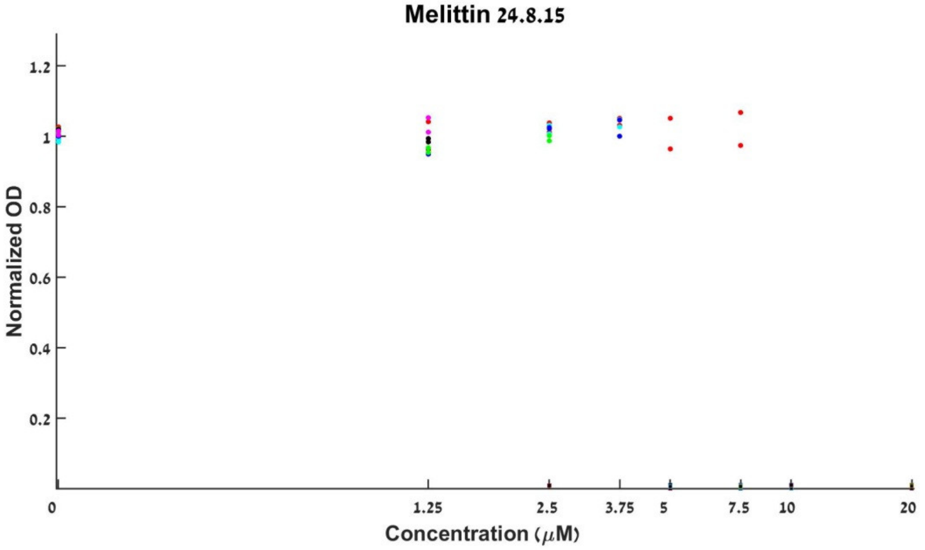
Melittin 24.8.15
Normalized OD
0.2
0.4
0.6
0.8
1
1.2
0
1.25
2.5
3.75
5
7.5
10
20
Concentration (μM)

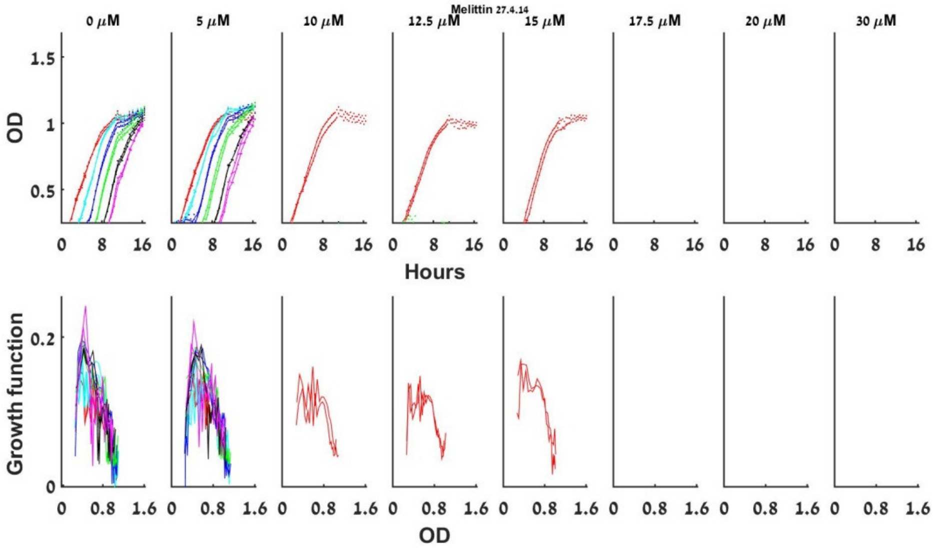
Melittin 27.4.14
0 μM
5 μM
10 μM
12.5 μM
15 μM
17.5 μM
20 μM
30 μM
OD
0.5
1
1.5
0
8
16
Hours
Growth function
0
0.2
0.8
1.6
OD

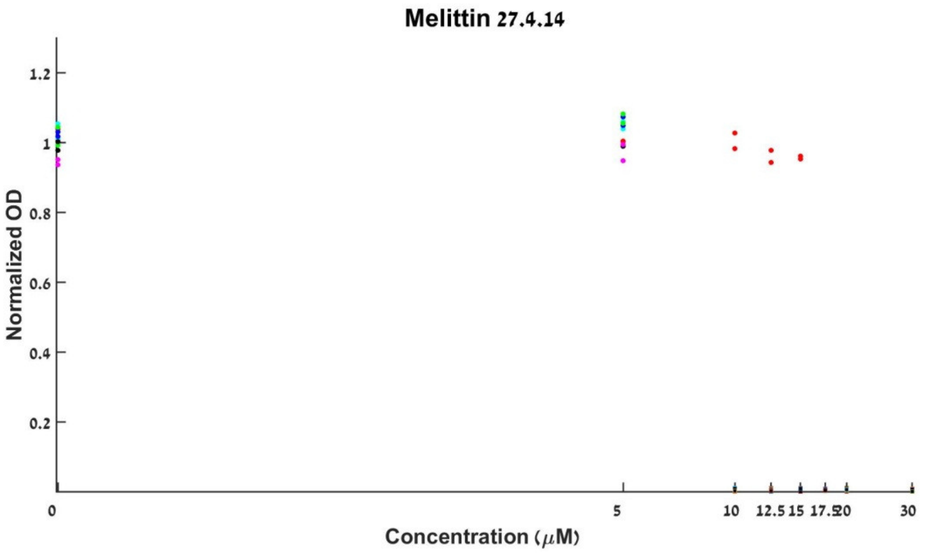

Figures S7D: K6L9 individual experiments

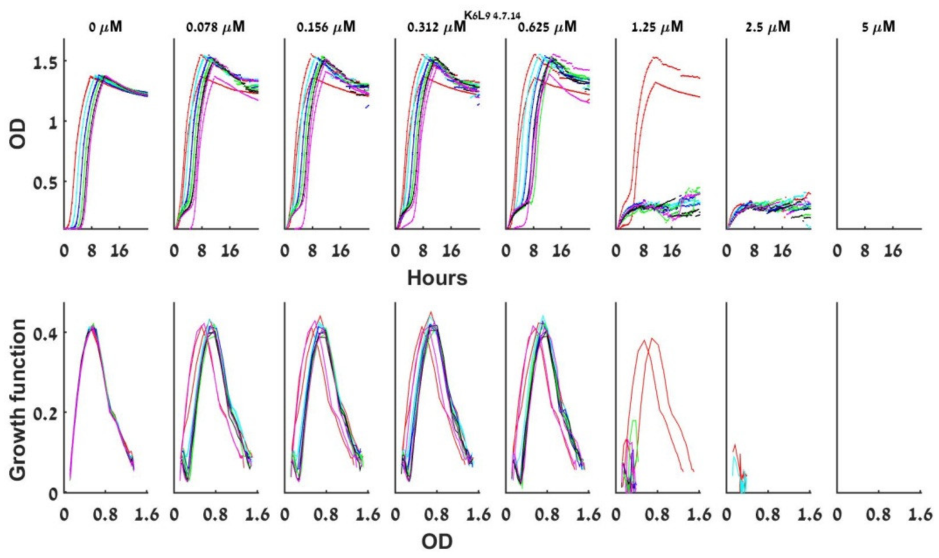

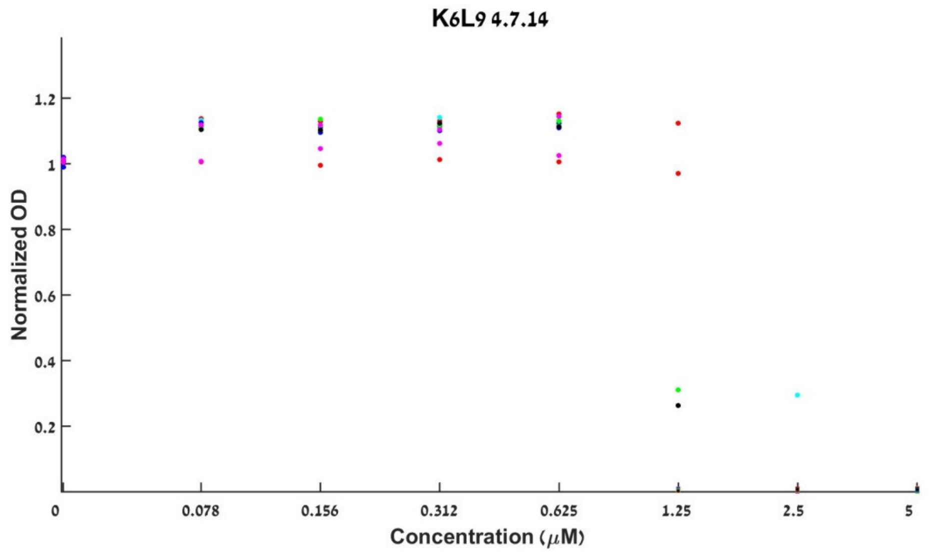
K6L9 4.7.14
Normalized OD
1.2
1
0.8
0.6
0.4
0.2
0
0.078
0.156
0.312
0.625
1.25
2.5
5
Concentration (μM)

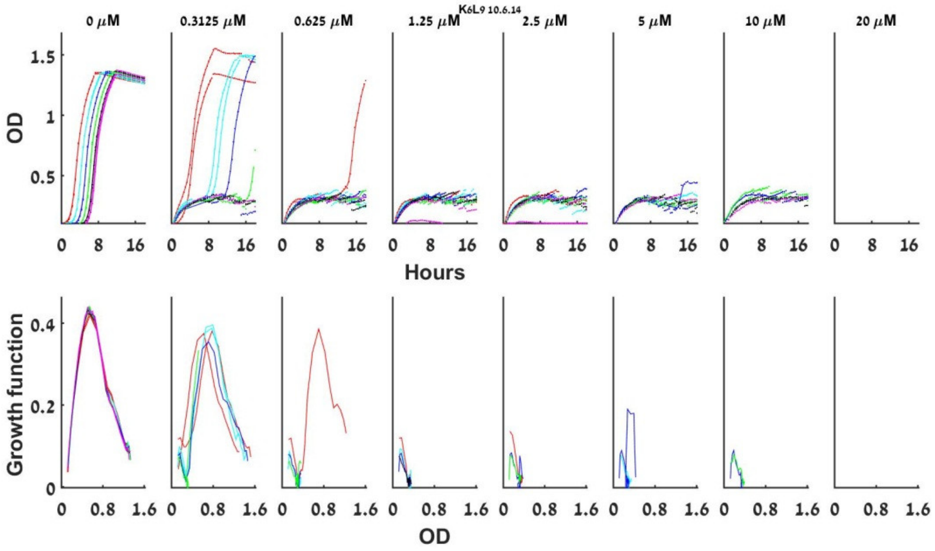
K6L9 10.6.14
0 μM
0.3125 μM
0.625 μM
1.25 μM
2.5 μM
5 μM
10 μM
20 μM
OD
1.5
1
0.5
0 8 16
Hours
Growth function
0.4
0.2
0
0 0.8 1.6
OD

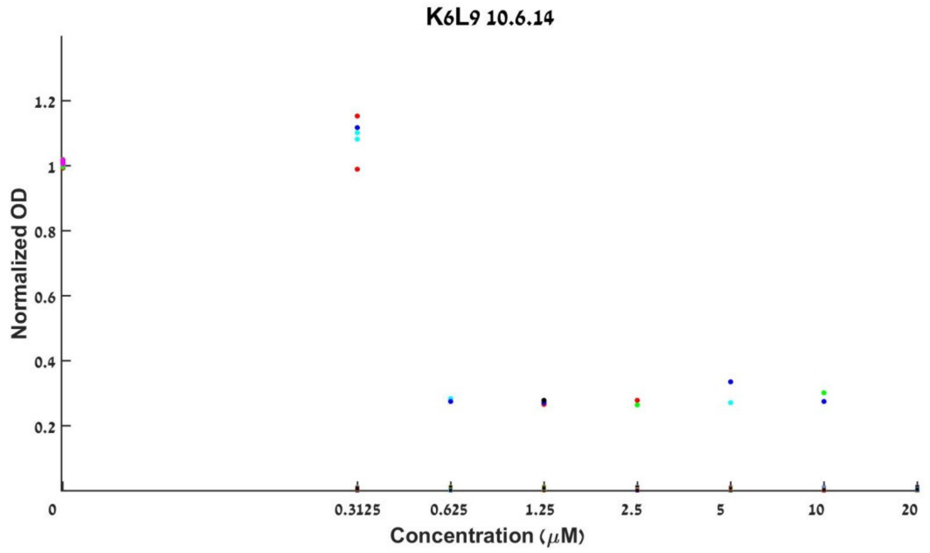
K6L9 10.6.14
Normalized OD
1.2
1
0.8
0.6
0.4
0.2
0
0.3125
0.625
1.25
2.5
5
10
20
Concentration (μM)

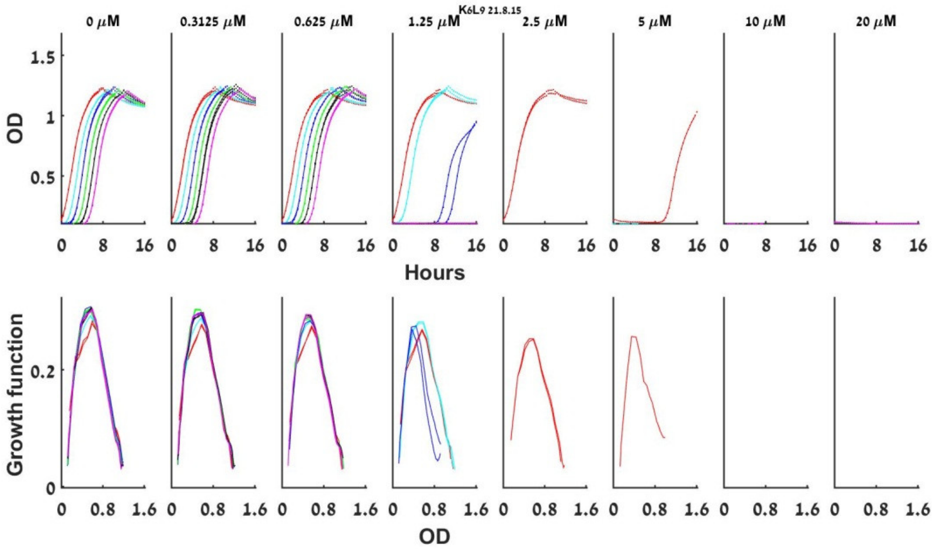
K6L9 21.8.15
0 μM
0.3125 μM
0.625 μM
1.25 μM
2.5 μM
5 μM
10 μM
20 μM
OD
Hours
Growth function
OD

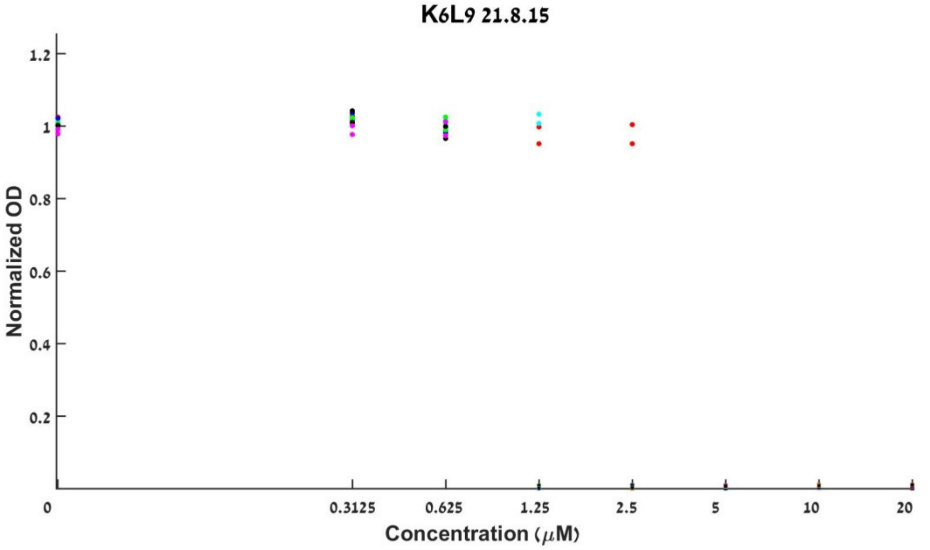
K6L9 21.8.15
Normalized OD
Concentration (μM)
0
0.3125
0.625
1.25
2.5
5
10
20

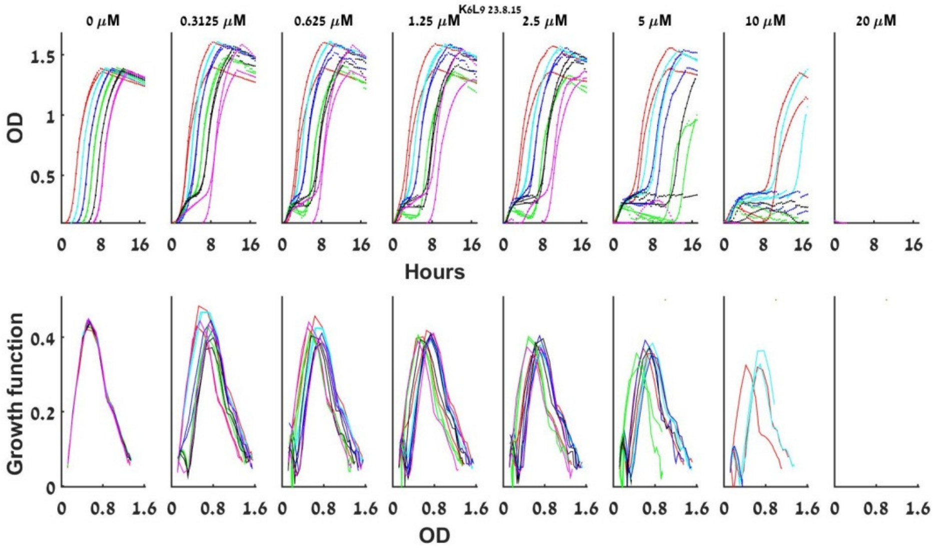
K6L9 23.8.15
0 μM
0.3125 μM
0.625 μM
1.25 μM
2.5 μM
5 μM
10 μM
20 μM
OD
Hours
Growth function
OD

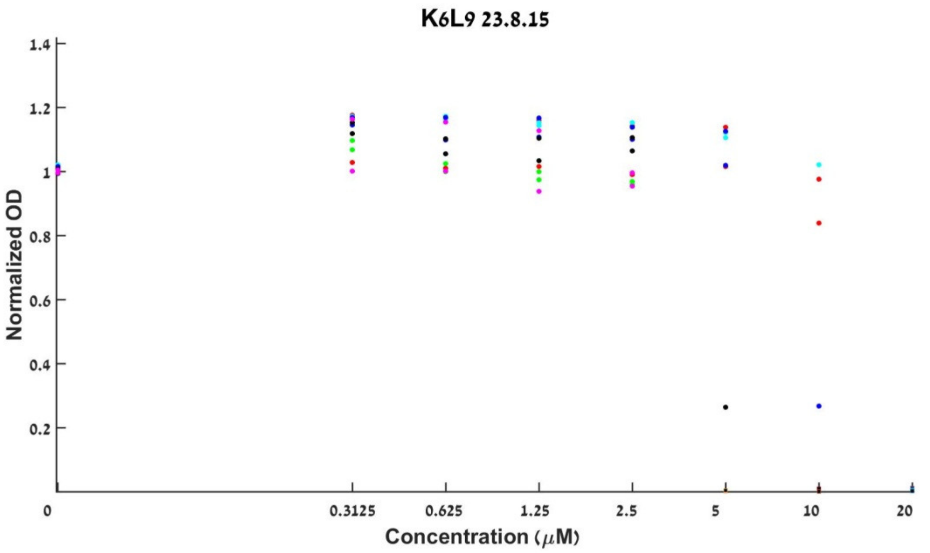
K6L9 23.8.15
Normalized OD
1.4
1.2
1
0.8
0.6
0.4
0.2
0
0.3125
0.625
1.25
2.5
5
10
20
Concentration (μM)

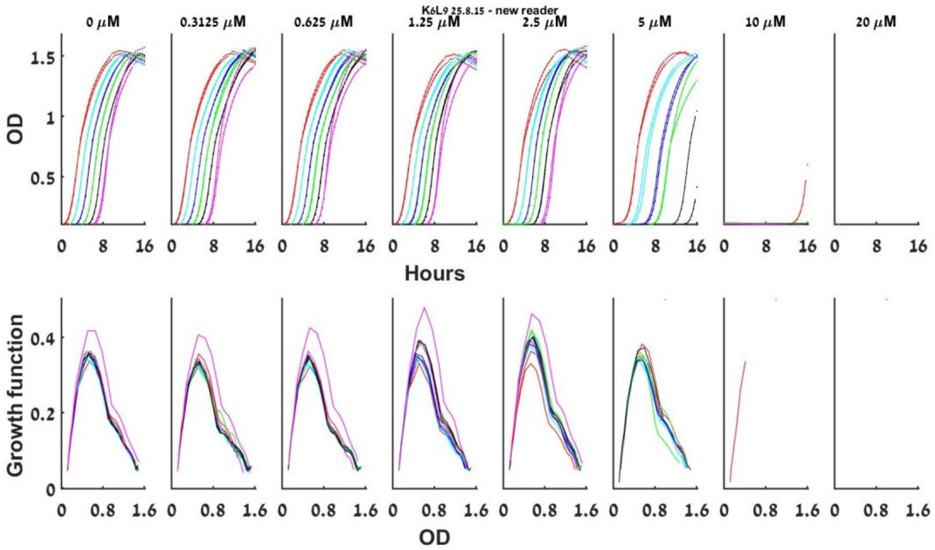
K6L9 25.8.15 - new reader
0 μM
0.3125 μM
0.625 μM
1.25 μM
2.5 μM
5 μM
10 μM
20 μM
OD
1.5
1
0.5
0 8 16
Hours
Growth function
0.4
0.2
0
0 0.8 1.6
OD

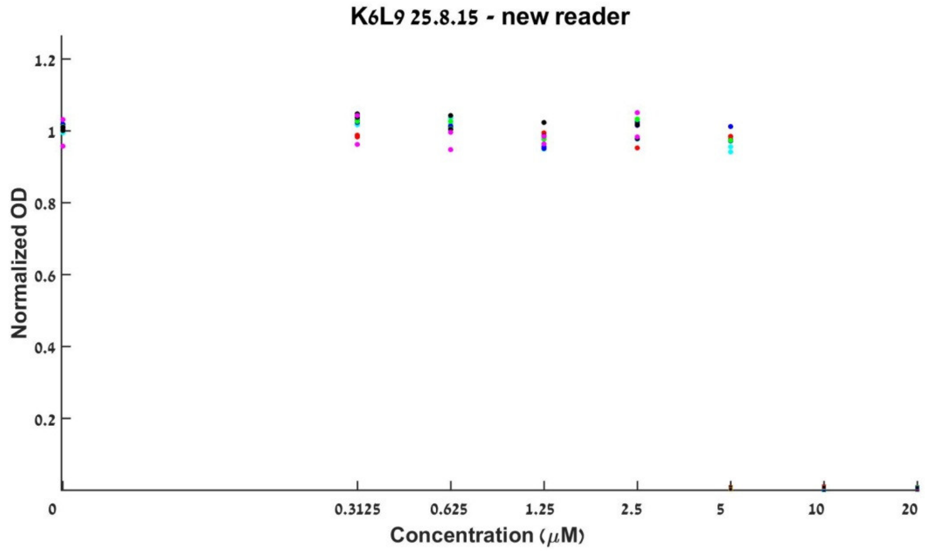
K6L9 25.8.15 - new reader
Normalized OD
1.2
1
0.8
0.6
0.4
0.2
0
0.3125
0.625
1.25
2.5
5
10
20
Concentration (μM)

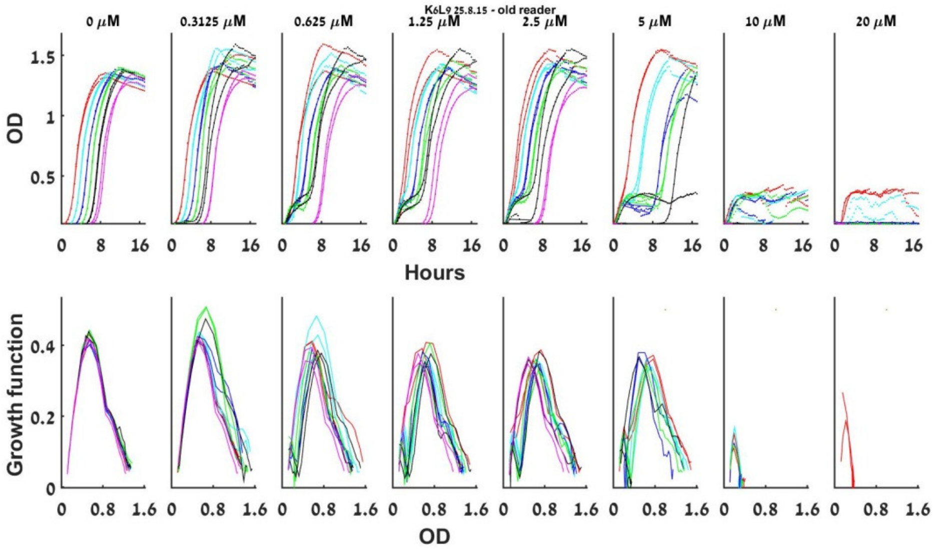
K6L9 25.8.15 - old reader
0 μM
0.3125 μM
0.625 μM
1.25 μM
2.5 μM
5 μM
10 μM
20 μM
OD
Hours
Growth function
OD

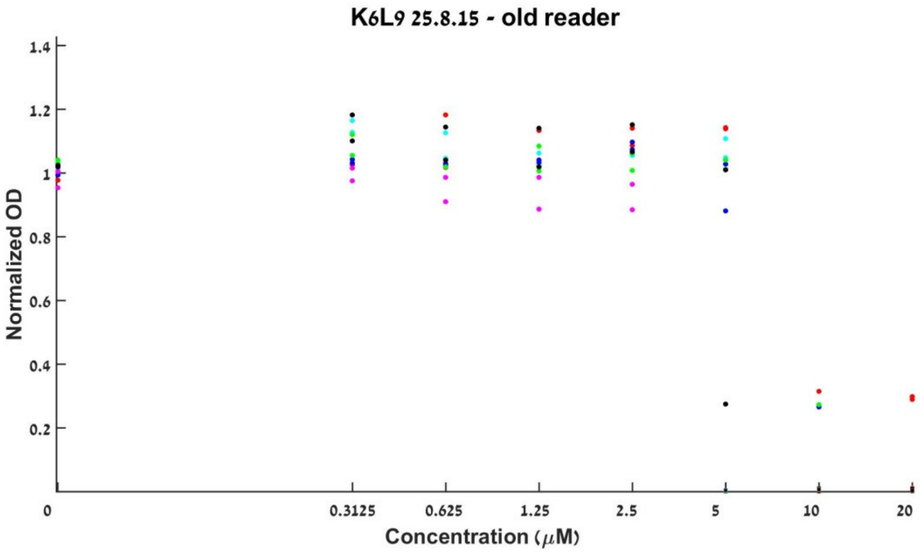
K6L9 25.8.15 - old reader
Normalized OD
Concentration (μM)

E

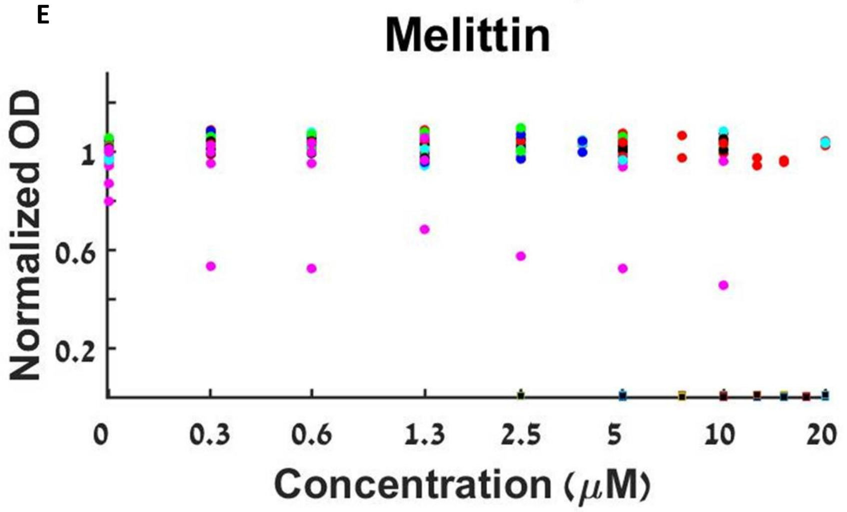

F

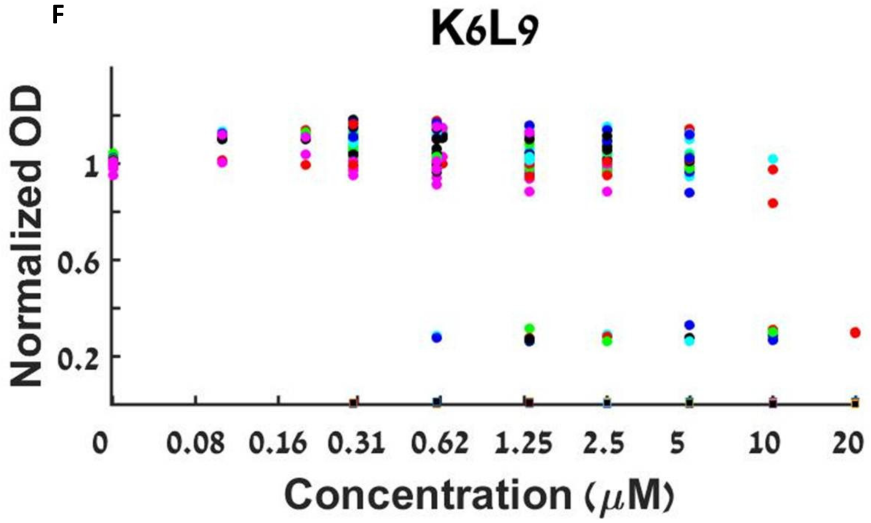

Figure S7. Bacterial growth dynamics under antimicrobial peptides treatment. Growth curves, growth functions and maximal capacity of single experiments performed with various antimicrobial peptides. (**A**) Single IE experiments with Polymixin B. (**B**) Single IE experiments with MSI. (**C**) Single IE experiments with Melittin. (**D**) Single IE experiments with K6L9. (**E**) Normalized maximal capacity of 6 IE experiments with Melittin (F) Normalized maximal capacity of 6 experiments with K6L9.
